# Transdiagnostic dimensions of psychopathology explain individuals’ unique deviations from normative neurodevelopment in brain structure

**DOI:** 10.1038/s41398-021-01342-6

**Published:** 2021-04-20

**Authors:** Linden Parkes, Tyler M. Moore, Monica E. Calkins, Philip A. Cook, Matthew Cieslak, David R. Roalf, Daniel H. Wolf, Ruben C. Gur, Raquel E. Gur, Theodore D. Satterthwaite, Danielle S. Bassett

**Affiliations:** 1 Department of Bioengineering, School of Engineering & Applied Science, University of Pennsylvania, Philadelphia, PA 19104 USA; 2 Department of Psychiatry, Perelman School of Medicine, University of Pennsylvania, Philadelphia, PA 19104 USA; 3 Lifespan Brain Institute, University of Pennsylvania & Children’s Hospital of Philadelphia, Philadelphia, PA 19104 USA; 4 Department of Radiology, Perelman School of Medicine, Philadelphia, PA 19104 USA; 5 Center for Biomedical Image Computing and Analytics, Perelman School of Medicine, University of Pennsylvania, Philadelphia, PA 19104 USA; 6 Department of Neurology, Perelman School of Medicine, Philadelphia, PA 19104 USA; 7 Department of Electrical & Systems Engineering, School of Engineering & Applied Science, University of Pennsylvania, Philadelphia, PA 19104 USA; 8 Department of Physics & Astronomy, College of Arts & Sciences, University of Pennsylvania, Philadelphia, PA 19104 USA; 9 Santa Fe Institute, Santa Fe, NM 87501 USA

**Keywords:** Predictive markers, Neuroscience, Psychology, Psychiatric disorders

## Abstract

Psychopathology is rooted in neurodevelopment. However, clinical and biological heterogeneity, together with a focus on case-control approaches, have made it difficult to link dimensions of psychopathology to abnormalities of neurodevelopment. Here, using the Philadelphia Neurodevelopmental Cohort, we built normative models of cortical volume and tested whether deviations from these models better predicted psychiatric symptoms compared to raw cortical volume. Specifically, drawing on the *p-factor* hypothesis, we distilled 117 clinical symptom measures into six orthogonal psychopathology dimensions: overall psychopathology, anxious-misery, externalizing disorders, fear, positive psychosis symptoms, and negative psychosis symptoms. We found that multivariate patterns of deviations yielded improved out-of-sample prediction of psychopathology dimensions compared to multivariate patterns of raw cortical volume. We also found that correlations between overall psychopathology and deviations in ventromedial prefrontal, inferior temporal, and dorsal anterior cingulate cortices were stronger than those observed for specific dimensions of psychopathology (e.g., anxious-misery). Notably, these same regions are consistently implicated in a range of putatively distinct disorders. Finally, we performed conventional case-control comparisons of deviations in a group of individuals with depression and a group with attention-deficit hyperactivity disorder (ADHD). We observed spatially overlapping effects between these groups that diminished when controlling for overall psychopathology. Together, our results suggest that modeling cortical brain features as deviations from normative neurodevelopment improves prediction of psychiatric symptoms in out-of-sample testing, and that *p-factor* models of psychopathology may assist in separating biomarkers that are disorder-general from those that are disorder-specific.

## Introduction

Throughout childhood, adolescence, and young adulthood, the brain undergoes major structural changes that facilitate the emergence of complex behavior and cognition^1,2^. Mental disorders often surface during this period^3^ and are increasingly understood as resulting from disruptions to normative brain maturation^4,5^. While maturational changes are stereotyped at the population level, substantial individual variation also exists^2^. The extent to which this individual variation in neurodevelopment may explain psychopathology remains unclear.

Linking abnormalities in neurodevelopment to psychopathology has been limited by several challenges. First, diagnostic nosologies assign individuals with distinct symptom profiles to the same clinical diagnosis, yielding disorder groups with highly heterogenous clinical presentation^6^. Second, comorbidity among disorders is high^7–9^, rendering it difficult to detect the neural correlates of specific disorders. Third, much of the extant literature has adopted case-control designs that reveal only abnormalities associated with the ‘average’ patient, ignoring the dimensional nature of psychopathology^10^. Research linking individuals’ neurodevelopmental alterations with distinct dimensions of psychopathology relevant to multiple disorders is a critical step toward developing diagnostic biomarkers for mental health^11–15^.

A promising approach entails examining dimensions of symptoms that cut across diagnostic categories^16^. The *p-factor* hypothesis^10,17–19^ posits that psychopathology symptoms cluster into latent dimensions including a general factor (known as *p* or ‘overall psychopathology’), which underpins individuals’ tendency to develop all forms of psychopathology, alongside multiple dimensions that describe specific types of psychopathology. This dimensional scoring can be accomplished with a bifactor model^20^, which yields specific factors (e.g., externalizing, psychosis) that are orthogonal to the general factor and to each other. Previous research has revealed that such psychopathology dimensions relate to differences in brain structure^19,21–24^. However, it remains unclear whether these psychopathology dimensions help to elucidate abnormal neurodevelopment; furthermore, it remains unknown to what extent they can help to dissociate disorder-general from disorder-specific abnormalities.

Here, we evaluated whether dimensions of psychopathology relate to individual differences in deviations from normative neurodevelopment. We modeled overall psychopathology and five specific factors, corresponding to mood and anxiety symptoms, externalizing behavior, fear, positive psychosis symptoms, and negative psychosis symptoms^21,25–27^. We integrated these psychopathology dimensions with T1-weighted neuroimaging data using a technique known as *normative modeling*^28^. Here, a normative model is a statistical model that finds the relationship between age and any brain feature, as well as the variation in this relationship expected in a group of healthy individuals. Then, the brains of individuals who experience psychopathology can be understood with respect to this normative model, allowing identification of regional deviations from normative neurodevelopment for each individual^29–31^. This approach allowed us to test whether individuals’ patterns of deviations from normative neurodevelopment were able to predict dimensions of psychopathology in out-of-sample testing. To examine the relative advantages of the normative model, we compared the predictive performance of deviations against the predictive performance of raw brain features.

The above framework is applicable to any brain feature that changes reliably with age. We focused on cortical gray matter, indexed via *cortical volume*, which is known to undergo plastic maturation in youth. Cortical volume shows a robust global decrease from childhood to adulthood, potentially reflecting cortical myelination and synaptic pruning^32–34^. Prior work has shown widespread non-uniform reductions in cortical volume—as well as thickness and surface area, which together comprise volume—across major depressive disorder^35,36^, schizophrenia^37^, bipolar disorder^38^, and anxiety disorders^39^. Across these disorders, overlapping reductions were particularly found in ventromedial prefrontal/medial orbitofrontal cortex (vmPFC/mOFC), inferior temporal, dorsal anterior cingulate (daCC), and insular cortices^39,40^.

Here, we sought to understand the link between dimensions of psychopathology from the *p-factor* model and deviations of cortical volume from patterns expected in normative neurodevelopment. We addressed three related questions. First, given that psychopathology may have neurodevelopmental origins, we tested the primary hypothesis that modeling cortical volume according to deviations from normative neurodevelopment would provide improved out-of-sample prediction of psychopathology compared to using raw volume.

Second, at the regional level, we expected that overall psychopathology would explain the common abnormalities observed in case-control studies^35–40^; we specifically predicted that greater overall psychopathology would be associated with greater negative deviations (i.e., lower than normative cortical volume) in the vmPFC/mOFC, inferior temporal, daCC, and insular cortices. Additionally, supporting the notion that effects in these regions predominantly represent disorder-general rather than disorder-specific biomarkers, we also predicted that correlations between deviations in these regions and overall psychopathology would be stronger than correlations between deviations and the specific dimensions in our model. We note that while it may be plausible to select other regions of interest to test this hypothesis, here our motivation was to select regions that have been robustly implicated across a broad range of distinct disorders. In accordance with this goal, the aforementioned regions were selected based on their consistent implication across multiple distinct clinical disorders in worldwide ENIGMA studies^35–38,40^ as well as meta-analyses^39^.

Third, we assessed the extent to which overall psychopathology explained the overlap between whole-brain group-level differences observed for traditional case-control analyses. Specifically, we examined group-level deviations from the normative model in two samples, one with depression and another with attention-deficit hyperactivity disorder (ADHD). We expected that both groups would show spatially correlated patterns of average deviations from normative neurodevelopment. Critically, we hypothesized that the correlation between patterns of average deviations would diminish when overall psychopathology was controlled for in our sample, indicating a lack of sensitivity of a case-control approach to detect disorder-specific biomarkers.

## Materials and methods

### Participants

The institutional review boards of both the University of Pennsylvania and the Children’s Hospital of Philadelphia approved all study procedures. Participants included 1601 individuals from the Philadelphia Neurodevelopmental Cohort^41^, a large community-based study of brain development in youths aged 8–22 years. All participants gave informed consent (for participants under the age of 18 years, assent and parental consent were obtained). All scanning was done using the same sequences on the same scanner without any hardware or firmware upgrades^41^. We studied a subset of 1271 participants, including individuals who were medically healthy and passed stringent quality control benchmarks for the neuroimaging data (see Supplementary Methods).

### Psychopathology dimensions

In this study, we take a transdiagnostic dimensional approach to assessing variation in the symptoms of mental health^11,13,16,42^. In particular, we use our recently published extended *p-factor* model^25^ based on the GOASSESS interview^26,43^. Briefly, the GOASSESS is an abbreviated and modified structured interview derived from the NIMH Genetic Epidemiology Research Branch Kiddie-SADS^44^ that covers a wide variety of psychiatric symptomatology such as the occurrence of mood (major depressive episode, mania), anxiety (agoraphobia, generalized anxiety, panic, specific phobia, social phobia, separation anxiety, obsessive-compulsive disorder), externalizing behavior (oppositional defiant, attention-deficit/hyperactivity, conduct disorder), and other behaviors. GOASSESS was administered by trained and certified assessors. The original model used a combination of exploratory and confirmatory factor analysis to distill the 112 item-level symptoms from the GOASSESS into five orthogonal dimensions of psychopathology. The original model included a factor common to all psychiatric disorders, referred to as overall psychopathology, as well as four specific factors: anxious-misery, psychosis, externalizing behaviors, and fear.

Here, as in our recent work^25^, we extended the above *p-factor* model in two ways. First, we included an additional five assessor-rated polytomous items (scored from 0 to 6, where 0 is ‘absent’ and 6 is ‘severe and psychotic’ or ‘extreme’, from the Scale of Prodromal Symptoms (SOPS) derived from the Structured Interview for Prodromal Syndromes (SIPS^45^) designed to measure the negative/disorganized symptoms of psychosis. These five items were (i) P5 disorganized communication, (ii) N2 avolition, (iii) N3 expression of emotion, (iv) N4 experience of emotions and self, and (v) N6 occupational functioning. Including this additional set of items brought the total number to 117. Second, we split the psychosis factor into two factors, one describing the delusions and hallucinations associated with the psychosis spectrum, which we hereafter refer to as psychosis-positive. The second psychosis factor described disorganized thought, cognitive impairments, and motivational-emotional deficits, which we hereafter refer to as psychosis-negative. We used confirmatory factor analysis implemented in Mplus^46^ to model five specific factors of psychopathology (anxious-misery, psychosis-positive, psychosis-negative, externalizing behaviors, and fear) as well as one common factor (overall psychopathology). See Table S1 for model statistics and Table S2 for factor loadings. Note, all phenotypes derived from this model are orthogonal to one another. To ensure normality, psychopathology dimensions were transformed using an inverse normal transformation^47,48^.

### Normative modeling

For details on image acquisition, processing, quality assurance (QA), and derivation of cortical volume, see Supplementary Methods. Briefly, cortical volume was extracted for each of 400 regions defined by the Schaefer atlas^49^. Next, we built normative models to predict regional volume. In order to estimate *normative* neurodevelopmental trajectories, we first split our sample of 1271 participants into two groups according to the presence or absence of psychiatric history. A total of 381 individuals reported no clinically significant symptoms on any disorder examined, while 890 individuals reported experiencing psychopathology. Next, in order to ensure that our analyses of deviations encompassed the full spectrum of psychopathology—including normal, subclinical, and clinical variance—we randomly sampled 100 of the 381 healthy individuals and combined them with the aforementioned 890 individuals to create a *testing* subset totaling 990 individuals. The remaining 281 healthy individuals were designated as our normative *training* subset. Next, for each brain region (*j*), we used gaussian process regression (GPR) to predict cortical volume values from age and sex using the training subset (see ref. ^28^ for details). An advantage of this approach is that in addition to fitting potentially nonlinear predictions of a brain feature, it also provides regional estimates of the expected variation in the relationship between age and brain features (normative variance) as well as estimates of uncertainty in this variance. Both normative variance and uncertainty are learned by the GPR from the training subset^28^. Then, for each participant (*i*) in the testing subset, we generated the predicted cortical volume ($\hat y_{ij}$) and combined it with the true cortical volume (*y*_*ij*_), the predictive uncertainty (*σ*_*ij*_), and the normative variance (*σ*_*nj*_) to create a *z*-score that quantified deviation from normative neurodevelopment^28^:

$$z_{ij} = \frac{{y_{ij} - \hat y_{ij}}}{{\sqrt {\sigma _{ij}^2 + \sigma _{nj}^2} }}$$

This normative model stands in contrast to alternative approaches, such as brain age models^50^, that typically use linear estimates of deviations in age rather than brain features and that do not account for normative variance and estimated uncertainty of deviations^51^. In the testing subset, the application of the normative model yielded a 990 × 400 *z*-score deviation matrix, ***Z***. Next, we used 10-fold cross-validation to also generate deviations in the training subset. The 281 individuals in the training subset were split into 10 folds, wherein 90% of the subset were used to re-train the GPR in order to generate *z*-score deviations in the remaining 10%. This process yielded a 281 × 400 *z*-score deviation matrix, ***Z***_cv_.

### Machine learning prediction models

First, we tested our primary hypothesis that scoring cortical volume as deviations from normative neurodevelopment would yield improved predictive performance compared to using raw volume values. Specifically, columns of ***Z*** were taken as multivariate input features to a ridge regression (RR, *α* = 1) to iteratively predict psychopathology dimensions (*y*) (see Fig. S1 for schematic representation). We performed 100 repeats^52^ of 10-fold cross-validation scored by root mean squared error (RMSE), mean absolute error (MAE), and the correlation between true and predicted *y*. Scoring metrics were averaged over folds. Note, to standardize the interpretation of all scoring metrics as higher scores represent better performance, we flip the sign for RMSE and MAE and examine negative RMSE and negative MAE. Within each fold, we applied principal component analysis (PCA) to reduce the dimensionality of ***Z*** to the 9 PCs that explained ≥1% variance in the data. Additionally, age and sex were controlled by regressing their effect out of *y* before predicting *y*. Nuisance regression was fit on the training data and applied to the test data to prevent leakage. To assess the significance of prediction performance, for each scoring metric, we averaged over the 100 repeats and compared the corresponding point estimate against a null distribution generated from a model trained on 100,000 random permutations of *y*. The *p*-values were assigned as the proportion of permuted scoring metrics that were greater than or equal to our true point estimates. Then, *p*-values were corrected for multiple comparisons over psychopathology dimensions via the Benjamini and Hochberg false discovery rate (FDR, *q* = 0.05) procedure^53^.

The above pipeline generated robust estimates of out-of-sample prediction performance that included significance testing. In order to directly compare predictive performance of normative deviations against raw cortical volume, we repeated this pipeline using a matrix of raw cortical volume values as input features. Note, analysis of raw volume was restricted to the testing subset to maintain equivalent statistical power, and the same number of PCs was used to maintain consistency in the number of input features. Together, for each psychopathology dimension and scoring metric, this process yielded two distributions of 100 performance values: one where prediction performance was measured using deviations from a normative model, estimated in an entirely independent sample (*training* subset, *n* = 281), and the other where performance was measured using raw cortical volume as predictors. Critically, as both models included identical nuisance regression applied to *y*, the only difference between models was in the way the cortical features were measured. For each psychopathology dimension and scoring metric, we compared prediction performance across input feature types using an exact test of differences^54^.

### Correlations between psychopathology dimensions and regional deviations from normative neurodevelopment

Our machine learning prediction model mapped the relationships between dominant sources of variance in cortical volume (deviations and raw) and psychopathology dimensions. Next, we examined our second hypothesis pertaining to the relative effect sizes of correlations between deviations and overall psychopathology and correlations between deviations and specific dimensions. First, we calculated Pearson correlations between each psychopathology dimension and deviations averaged over subsets of Schaefer parcels that comprised the vmPFC/mOFC, inferior temporal, daCC, and insular areas (see Supplementary Methods). Next, for each region (e.g., daCC), we tested for differences in effect sizes (Pearson’s *r*) between overall psychopathology and each specific psychopathology dimension using bootstrapping. Specifically, in each of 10,000 bootstrapped samples, we subtracted the absolute effect size for each specific dimension from the absolute effect size for overall psychopathology. Then, upon these distributions of effect size differences, we calculated the 99% confidence interval (CI) and considered overall psychopathology to have yielded a significantly stronger effect if the lower CI bound was >0 (this point corresponded to a Bonferroni-corrected threshold of *p* < 0.01). Second, we supplemented this analysis with a whole-brain mass univariate analysis, wherein Pearson correlations were calculated between each psychopathology dimension and each column in ***Z*** (i.e., Schaefer parcels). Here, multiple comparisons were corrected across parcels and psychopathology dimensions (2400 tests) using the Benjamini and Hochberg false discovery rate (FDR, *q* = 0.05) procedure^53^ (see Supplementary Methods).

### Case-control comparisons of deviations from normative neurodevelopment

Finally, we tested our third hypothesis that group-level effects derived from case-control designs would be confounded by insensitivity to disorder-specific effects. We selected a subsample of our testing subset with clinically significant depression (*n* = 136, mean age = 17.57 ± 2.3 years, 32% males) and a subsample with clinically significant ADHD (*n* = 163, mean age = 13.62 ± 3.11, 63% males), two disorders with distinct clinical presentations, and performed case-control analyses. In each group, we excluded participants with comorbid depression and ADHD. Next, we selected two subsamples of HCs from our full HC cohort (i.e., *n* = 381) that were matched to each of our patient groups on age, sex, T1 QA, and T1 signal-to-noise ratio (SNR). This procedure yielded two independent subgroups of HCs. Then, we estimated group-level deviations from normative neurodevelopment by calculating regional Cohen’s *d* values comparing the deviations from each patient group with their matched HC counterparts. This process not only minimized the effects of the aforementioned confounds, but it also ensured that the Cohen’s *d* maps for each patient group were estimated independently from one another. Then, to assess the correspondence between group-level effects for depression and ADHD, we estimated the spatial (Pearson’s) correlation between regional Cohen’s *d* values. Finally, to examine the extent to which regional variation in Cohen’s *d* values was explained by overall psychopathology, we re-estimated the spatial correlation between regional Cohen’s *d* values after controlling for overall psychopathology. Note, control of overall psychopathology was conducted independently for each patient group.

## Results

### Participants

Sample demographics, including counts of individuals who met diagnostic criteria for lifetime presence of a broad array of disorders, are shown in Table 1 (see also Fig. S2 for mean symptom dimensions as a function of diagnostic groups). Figure S3 shows that males in our sample had significantly higher scores on psychosis-positive (*t* = 2.30, *p* < 0.05_FDR_), psychosis-negative (*t* = 3.84, *p* < 0.05_FDR_), and externalizing (*t* = 4.84, *p* < 0.05_FDR_) factors. Females had significantly higher scores on anxious-misery (*t* = −4.34, *p* < 0.05_FDR_) and fear (*t* = −5.88, *p* < 0.05_FDR_) factors. No significant effect of sex was observed for overall psychopathology. Figure S4 shows that age correlated significantly with higher overall psychopathology (*r* = 0.32, *p* < 0.05_FDR_) and lower externalizing (*r* = −0.09, *p* < 0.05_FDR_) scores. Finally, see Fig. S5 for characterization of trajectories derived from the normative models (i.e., age effects on cortical volume as a function of sex).

**Table 1:** Summary of demographic and psychopathology data.

	Sample	Training subset	Testing subset
	(*n* = 1271)	(*n* = 281)	(*n* = 990)
Age, year, mean (±SD)	15.10 (±3.64)	14.79 (±3.97)	15.13 (±3.54)
Sex, *n* (%)			
Male	603 (47.44)	146 (51.96)	457 (46.16)
Female	668 (52.56)	135 (48.04)	533 (53.84)
Psychopathology categories, *n* (%)			
Psychosis spectrum	364 (28.64)	0 (0)	364 (28.64)
Manic episode	13 (1.02)	0 (0)	13 (1.02)
Major depressive episode	179 (14.08)	0 (0)	179 (14.08)
Bulimia	5 (0.39)	0 (0)	5 (0.39)
Anorexia	16 (1.26)	0 (0)	16 (1.26)
Social anxiety disorder	295 (23.21)	0 (0)	295 (23.21)
Panic	13 (1.02)	0 (0)	13 (1.02)
Agoraphobia	73 (5.74)	0 (0)	73 (5.74)
Obsessive compulsive	41 (3.23)	0 (0)	41 (3.23)
Post-traumatic stress	156 (12.27)	0 (0)	156 (12.27)
Attention deficit hyperactivity	206 (16.21)	0 (0)	206 (16.21)
Oppositional defiant	407 (32.02)	0 (0)	407 (32.02)
Conduct	102 (8.03)	0 (0)	102 (8.03)

Owing to comorbidity, individual participants may be present in more than 1 category of lifetime prevalence. All individuals who met criteria for lifetime prevalence were in the testing subset.

### Modeling deviations from normative neurodevelopment improves the prediction of dimensions of psychopathology in out-of-sample testing

**Fig. 1:**
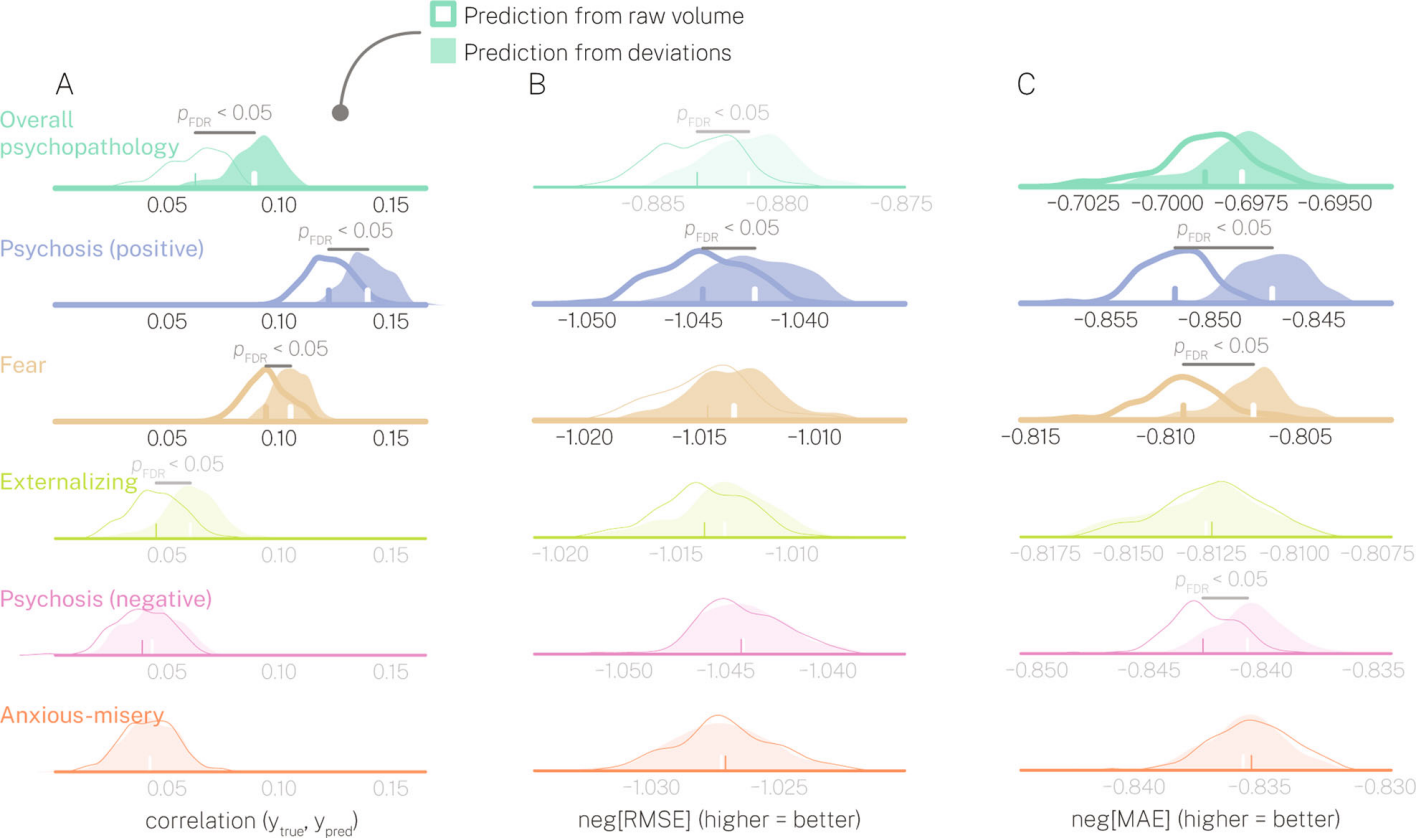
Deviations from normative neurodevelopment yield improved predictive performance of overall psychopathology, positive psychosis, and fear symptoms in out-of-sample testing. Predictive performance for each of six dimensions of psychopathology (rows) as a function of multiple scoring metrics (columns **A**–**C**). In each subplot two distributions are presented: one that illustrates predictive performance derived from raw cortical volume (white distribution with colored outline), and one that illustrates predictive performance derived from deviations from normative models (colored distribution). Distributions of predictive performance that did not yield above chance performance are shown with partial transparency and lighter stroke. Predictive performance for overall psychopathology, positive psychosis, and fear was above chance levels and are shown with heavier stroke. The scoring metrics based on model error (i.e., RMSE, MAE) are shown with a negative sign so that higher values equal better performance across all scoring metrics. Thus, neg[RMSE] = negative root mean squared error, and neg[MAE] = negative mean absolute error. Differences in scoring metrics between raw cortical volume and deviations were assessed using an exact test of differences. Significant effects are marked with *p* < 0.05_FDR_.

To address our primary hypothesis that examining deviations from normative models of neurodevelopment would improve predictive performance of dimensions of psychopathology, we compared cross-validated out-of-sample prediction performance when cortical features were scored using deviations to when cortical features were scored using raw volume values (see Figs. S6, S7, S8 for illustration of the principal components that were used as input features for prediction). First, our permutation test revealed that we were only able to predict overall psychopathology (see Fig. S9 for prediction as a function of individual PCs), psychosis-positive, and fear above chance levels (Fig. 1). Critically, for these dimensions, using deviations yielded comparable or better predictive performance compared to using raw volume across multiple scoring metrics. For example, deviations yielded significantly higher correlations between true and predicted *y* (Fig. 1a), significantly higher negative RMSE (Fig. 1b) for overall psychopathology and psychosis-positive, as well as significantly higher negative MAE for psychosis-positive and fear (Fig. 1c). Furthermore, even for dimensions where predictive performance was not above chance levels (i.e., externalizing, psychosis-negative, anxious-misery), we still observed instances where deviations yielded significantly better predictive performance. For example, deviations yielded significantly higher correlations between true and predicted *y* for externalizing (Fig. 1a) and significantly higher negative MAE for psychosis-negative (Fig. 1c). In fact, we never observed a psychopathology dimension where (i) raw volume yielded significantly better predictive performance compared to deviations, or (ii) where raw volume yielded above-chance predictive performance, but deviations did not. Thus, in partial support of hypothesis 1, our results demonstrate that modeling deviations from normative neurodevelopment provided better out-of-sample prediction of overall psychopathology, psychosis-positive, and fear compared to using raw cortical volume (see Fig. S10 showing that externalizing and psychosis-negative scores could be predicted above chance levels when excluding individuals on psychoactive medication). Finally, see Figs. S11–15 for a series of sensitivity analyses spanning an extensive set of additional nuisance covariates, including years of maternal education, indices of T1 QA, and general intelligence.

### Psychopathology dimensions explain regional deviations from normative neurodevelopment

Next, we tested our second hypothesis pertaining to the relationships between psychopathology dimensions and deviations from normative neurodevelopment in our a priori regions of interest. First, as expected, we found that overall psychopathology correlated significantly with deviations in the vmPFC/mOFC (*r* = −0.15, *p* < 0.05_FDR_), inferior temporal (*r* = −0.15, *p* < 0.05_FDR_), daCC (*r* = −0.12, *p* < 0.05_FDR_), and insular cortices (*r* = −0.11, *p* < 0.05_FDR_), wherein greater scores corresponded to greater negative deviations. Additionally, the psychosis-positive dimension significantly correlated with deviations in the inferior temporal cortex (*r* = −0.08, *p* < 0.05_FDR_) and daCC (*r* = −0.07, *p* < 0.05_FDR_). Finally, the fear dimension significantly correlated with deviations in the vmPFC/mOFC (*r* = −0.07, *p* < 0.05_FDR_), inferior temporal (*r* = −0.11, *p* < 0.05_FDR_) and insular cortices (*r* = −0.13, *p* < 0.05_FDR_). Notably, the psychosis-negative, anxious-misery, and externalizing dimensions did not yield significant correlations to deviations in any of our regions of interest.

**Fig. 2:**
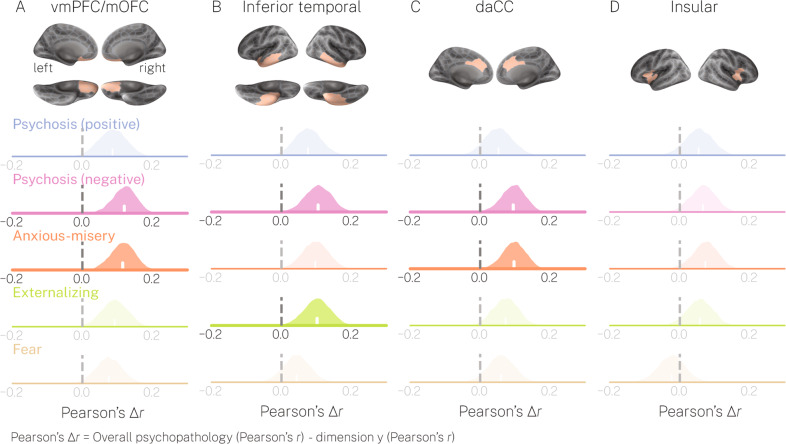
Correlations between overall psychopathology and deviations from normative neurodevelopment are stronger than correlations observed for specific dimensions of psychopathology. In each subplot, distributions of absolute Pearson’s correlation coefficients between each specific psychopathology dimension (rows) and regional deviations (columns **A**–**D**) were subtracted from absolute correlations observed for overall psychopathology in the same region. Note, Pearson’s correlations were calculated after residualizing both psychopathology dimensions and deviations with respect to T1 QA and T1 SNR (see Supplementary Methods for details). Performing this subtraction 10,000 times across bootstrapped samples generated distributions of effect size differences, ∆*r*. Positive ∆*r* indicates that correlations for overall psychopathology were greater when compared to those observed for the specific dimensions. The ∆*r* distributions for which the lower bound of the 99% confidence interval was >0 are shown with heavier stroke and no transparency.

Second, we directly compared the effect sizes between those observed for overall psychopathology and those observed for each specific dimension (Fig. 2; Pearson’s ∆*r*). We found that, for most of our regions of interest, correlations between deviations and overall psychopathology were larger (average ∆*r* > 0) than those observed for specific psychopathology dimensions. The only exception was for the comparison with the fear dimension in the insular cortex, where Pearson’s ∆*r* was approximately 0. These positive ∆*r* values were significantly >0 in the vmPFC/mOFC for the psychosis-negative and anxious-misery dimensions, in the inferior temporal cortex for the psychosis-negative and externalizing dimensions, as well as in the daCC for the psychosis-negative and anxious-misery dimensions. In each of these cases, correlations between deviations and overall psychopathology were significantly larger than those observed for the other psychopathology dimensions at the *p* < 0.01 level. At a more relaxed threshold of *p* < 0.05 (i.e., lower bounds of 95% CI > 0), positive ∆*r* values were significantly >0 in the vmPFC/mOFC for all psychopathology dimensions, in the inferior temporal cortex for all dimensions except fear, in the daCC for the psychosis-negative, anxious-misery, and externalizing dimensions, as well as in the insular cortex for the anxious-misery dimension (Fig. S16). Thus, in partial support of our second hypothesis, these results demonstrate that abnormalities in regions commonly reported in the case-control literature may be predominantly associated with overall psychopathology.

**Fig. 3:**
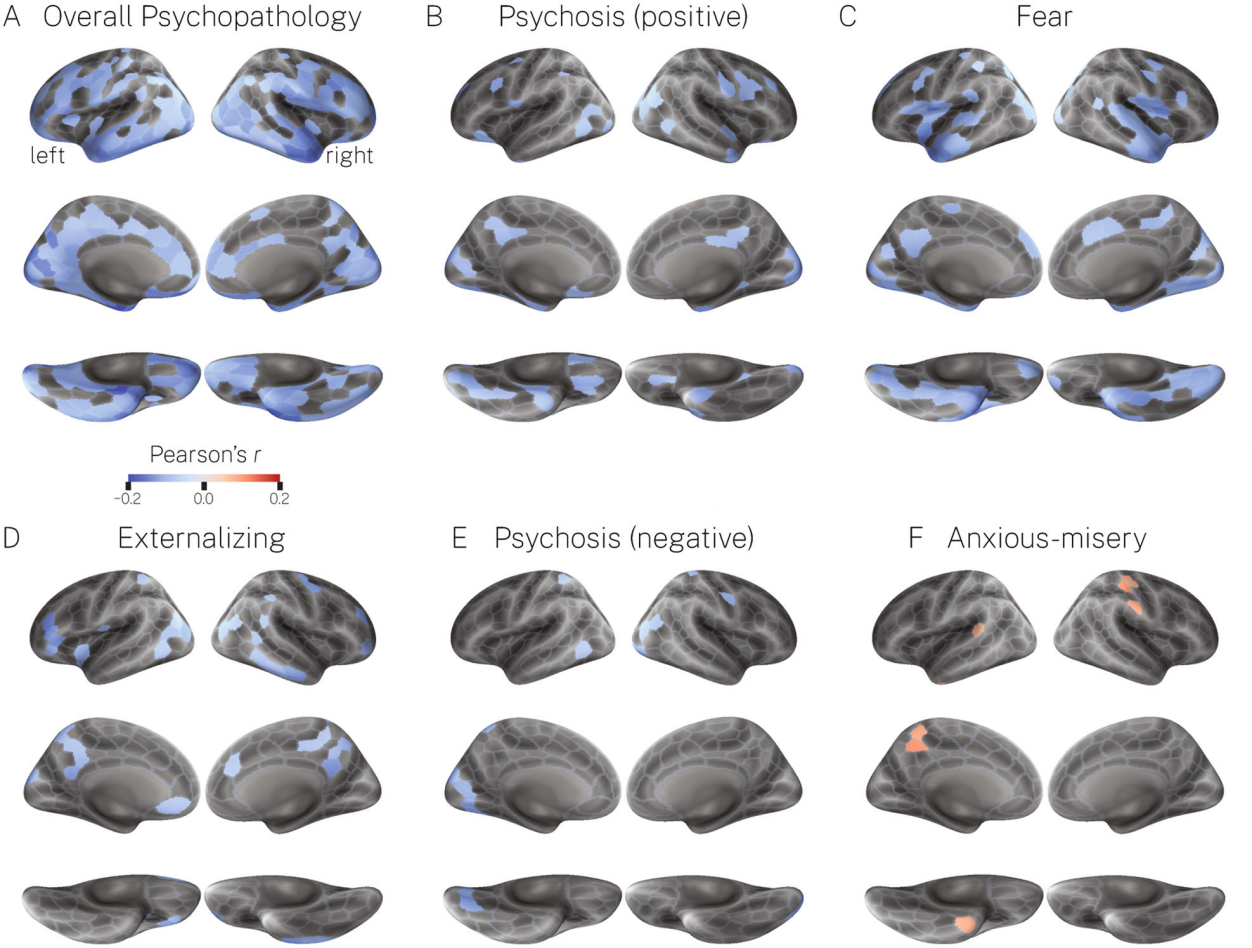
The bivariate relationship between dimensions of psychopathology and deviations from normative neurodevelopment for cortical volume. **A**–**F** Significant Pearson’s correlation coefficients between dimensions of psychopathology and deviations from the normative model. For negative correlations, greater scores on the psychopathology dimension are associated with greater *negative* deviations from normative neurodevelopment. For positive correlations, greater scores on the psychopathology dimension are associated with greater *positive* deviations from normative neurodevelopment (note: this only occurred for anxious-misery; see panel **F**).

Consistent with the above findings, our mass univariate analysis of the 400 Schaefer parcels revealed that greater scores on overall psychopathology was associated with widespread negative deviations across the cortex, including in the vmPFC/mOFC, inferior temporal cortex, daCC, and the insular cortex, albeit to a lesser extent (Fig. 3a). Furthermore, compared to overall psychopathology, significant correlations for the specific psychopathology dimensions were relatively sparse and infrequently occurred in our a priori regions of interest with the exception of fear (Fig. 3b–f). Finally, our mass univariate analysis revealed a notable consistency in our results, which was that overall psychopathology, fear, and to a lesser extent psychosis-positive, psychosis-negative, and externalizing, each showed relatively widespread effects in the visual and somatomotor systems.

### Group effects yield non-specific patterns of deviations from normative neurodevelopment

**Fig. 4:**
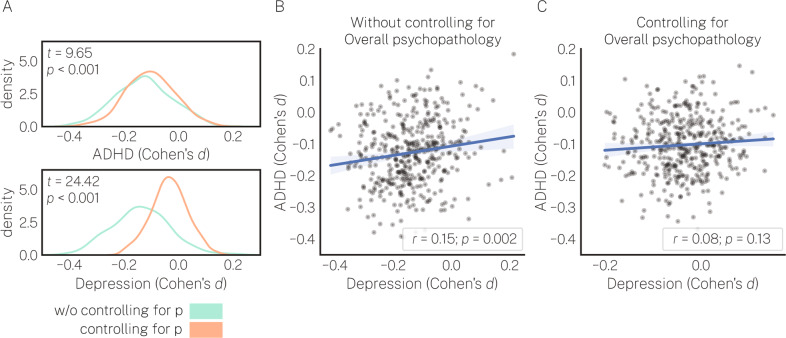
Deviations from normative neurodevelopment in depression and ADHD groups show correlated whole-brain effects confounded by overall psychopathology. Case-control comparisons were conducted examining group differences in deviations between individuals with depression and individuals with ADHD compared to independent samples of healthy individuals matched on age, sex, T1 QA, and T1 SNR. **A** Regional Cohen’s *d* values from the ADHD group (top) and depression group (bottom) with and without controlling for overall psychopathology. For both groups, controlling for overall psychopathology resulted in a significant shift in Cohen’s *d* values towards zero. **B** Regional Cohen’s *d* values from the depression group correlate with regional Cohen’s *d* values from the ADHD group. **C** Correlations between depression and ADHD groups decrease when controlling for overall psychopathology.

Above, we demonstrated that overall psychopathology tracked variation in deviations from normative neurodevelopment in regions commonly implicated in the case-control literature. Next, we examined the extent to which overall psychopathology accounted for the spatial overlap between group-level differences observed for case-control analyses conducted in two specific disorders that are conceptualized as dissimilar: depression and ADHD. Figure 4a shows the distribution of Cohen’s *d* values for ADHD and depression for cortical volume with and without controlling for overall psychopathology. For both groups, controlling for overall psychopathology resulted in a significant shift in the Cohen’s *d* distribution towards zero (ADHD, *t* = 9.65, *p* < 0.001; depression, *t* = 24.42, *p* < 0.001). Figure 4b shows the relationship between regional Cohen’s *d* values in each group without controlling for overall psychopathology (*r* = 0.15, *p* = 0.002), while Fig. 4c shows the relationship between regional Cohen’s *d* when controlling for the effect of overall psychopathology (*r* = 0.08, *p* = 0.13). Notably, controlling for overall psychopathology reduced the correlation between depression and ADHD to *r* *=* 0.08; a ∆ of 0.07. We repeated this analysis using each of the other psychopathology dimensions and found that this relationship was specific to overall psychopathology; when controlling for other dimensions, the spatial correlation ∆ was, on average, 0.006. Together, these results suggest that the spatial correspondence between group-level deviations for two clinically dissimilar disorders was explained by overall psychopathology.

## Discussion

Mental disorders are increasingly viewed as disorders of neurodevelopment^3–5,55^. However, heterogeneity in both neurodevelopmental trajectories and symptom profiles have confounded case-control designs and made it difficult to precisely characterize the relationship between abnormalities in neurodevelopment and the symptoms of psychopathology. Here, we showed that modeling cortical volume as deviations from normative models of neurodevelopment improved the prediction of overall psychopathology, psychosis-positive and fear dimensions in out-of-sample testing. Furthermore, at the regional level, we demonstrated that overall psychopathology correlated with greater negative deviations in vmPFC/mOFC, inferior temporal, daCC, and insular cortices—all regions previously implicated in case-control literature across a broad spectrum of disorders^35–40^—and that these correlations were, in some cases, significantly larger than those observed for the psychosis-negative, anxious-misery, and externalizing dimensions. Finally, we found that case-control comparisons between two clinically dissimilar groups (depression and ADHD) and healthy controls showed spatially correlated group differences in deviations that diminished when controlling for overall psychopathology, suggesting that overall psychopathology confounded case-control comparisons. Overall, our results demonstrate that the combination of normative models of neurodevelopment and *p-factor* models of psychopathology not only have the potential to improve prediction of the symptoms of mental disorder but may also help to tease apart disorder-general from disorder-specific biomarkers in psychiatry.

Previous studies have revealed non-uniform gray matter reductions concentrated in vmPFC/mOFC, inferior temporal, daCC and insular cortices across major depressive, bipolar, schizophrenia, and anxiety disorders^35–40^. Here, our analysis of our a priori regions of interest and our mass univariate analysis indicated that deviations in each of these regions were implicated predominantly, and in some cases most strongly, by overall psychopathology. The main exception was the insular cortex, where correlations between overall psychopathology and deviations were not significantly larger than those observed for the specific dimensions in our model. Together, our findings suggest that the effects commonly reported in the case-control literature pertaining to the vmPFC/mOFC, daCC, and inferior temporal cortices may reflect the general neural correlates of mental disorder more than disorder-specific signatures^39^, and that the use of *p-factor* models may assist psychiatry researchers in separating the two.

Beyond our a priori regions of interest, our mass univariate analysis revealed that regions in the visual and somatomotor systems were broadly impacted by overall psychopathology. This observation is consistent with recent functional connectivity work^56,57^. Elliot et al*.*^56^ showed that overall psychopathology correlated with dysconnectivity between the visual systems and the frontoparietal and default mode systems, and Kebets et al.^57^ showed that overall psychopathology correlated with dysconnectivity within and between somatomotor and visual systems. Although there are several clear differences between the research of Elliot et al*.* and Kebets et al. and the current study, including neuroimaging modality, clinical assessments, statistical methodology, and sample age, the results converge on the idea that disruptions to lower-order brain systems may be common across mental disorders. Indeed, given that our sample was, on average, younger than the sample used by Elliot et al. (which in turn was younger than that used by Kebets et al.), our results suggest that these markers of disorder-general pathophysiology may emerge early during disease progression and persist into adulthood. Datasets covering the lifespan will be critical to testing precise developmental timing effects associated with visual and somatomotor pathophysiology, including the relationship between brain structure and function.

### Limitations

A limitation of this study is the use of cross-sectional data to model neurodevelopment. It is well documented that individual variability in neurodevelopment occurs at both the inter-individual and intra-individual level^2^, and characterizing the factors that explain the latter will be critical for predicting the emergence of psychopathology over time. Thus, future work should test whether the brain regions identified using our approach explain variance in psychopathology dimensions at follow-up time points. Another limitation is the focus on T1-weighted cortical brain features. The underlying white matter pathways change throughout development in parallel with the cortex, giving rise to segregated processing modules that increase the functional efficiency of the brain^58–60^. Here, we focused on cortical volume derived from T1-weighted imaging data owing to the robust relationship with age, which is well suited to normative modeling. Future work that builds multimodal normative models of neurodevelopment may provide additional insights into the physiology of psychopathology, which is critical to progressing the field of psychiatry towards personalized medicine.

Finally, we note that controlling for socioeconomic status and general intelligence in our predictive models resulted in many of our prediction models dropping below significance under our permutation test (see Supplementary Materials), suggesting that these measures confounded our prediction models. However, we note that predictive performance of overall psychopathology from deviations was still significantly higher than from raw brain volume, suggesting that the benefit of using the normative model persisted. Nevertheless, this result suggests future work should examine the extent to which socioeconomic status and general intelligence might mediate the relationship between deviations from normative neurodevelopment and psychopathology.

## Conclusions

Our results represent an important step toward understanding the link between neurodevelopment and psychopathology. We explicitly modeled normative variance in neurodevelopment, allowing us to estimate multivariate continuous single-subject neurodevelopmental abnormalities. Combining this approach with a dimensional model of psychopathology allowed us to improve the out-of-sample prediction of psychiatric symptoms compared to raw cortical volume, particularly for overall psychopathology. This result provides proof of principle that recasting structural neuroimaging data obtained from one sample as deviations from a separate normative cohort can improve the predictive relationship between brain and symptom data. This is encouraging because it supports the viability of generating standardized normative models that may then be deployed to understand variance in brain data acquired across multiple different clinical sites. However, this interpretation remains speculative, since our analysis was restricted to a single site. In order to examine whether the improved predictive performance observed here generalizes to new samples, future work should consider using a normative model trained on one site to understand variance in brain data acquired from a different site. Indeed, new methods from normative modeling are being developed that may facilitate this goal^61^.

Our work also underscores the importance of decoupling specific forms of psychopathology from overall psychopathology. Not doing so may confound the capacity of case-control designs to discover disorder-specific signatures of abnormal neurodevelopment. This confound, in turn, renders case-control designs less likely to yield clinically useful biomarkers in psychiatry. Finally, our work contributes to a growing body of literature demonstrating that, in order to discover neurodevelopmental biomarkers for mental health, psychiatric research could benefit from supplementing examination of the statistical ‘average patient’ with dimensional approaches to psychopathology and brain pathophysiology^14,15,28^. Such a neurobiologically-grounded framework may provide a step towards personalized medicine in psychiatry, and ultimately allow for the improved outcome for patients.

### Citation Diversity Statement

Recent work in several fields of science has identified a bias in citation practices such that papers from women and other minority scholars are under-cited relative to the number of such papers in the field^62–66^. Here we sought to proactively consider choosing references that reflect the diversity of the field in thought, form of contribution, gender, race, ethnicity, and other factors. First, we obtained the predicted gender of the first and last author of each reference by using databases that store the probability of a first name being carried by a woman^66,67^. By this measure (and excluding self-citations to the first and last authors of our current paper), our references contain 15.19% woman(first)/woman(last), 16.24% man/woman, 22.1% woman/man, and 46.46% man/man. This method is limited in that (a) names, pronouns, and social media profiles used to construct the databases may not, in every case, be indicative of gender identity and (b) it cannot account for intersex, non-binary, or transgender people. Second, we obtained predicted racial/ethnic category of the first and last author of each reference by databases that store the probability of a first and last name being carried by an author of color^68,69^. By this measure (and excluding self-citations), our references contain 13.06% author of color (first)/author of color(last), 22.88% white author/author of color, 18.62% author of color/white author, and 45.44% white author/white author. This method is limited in that (a) names and Wikipedia profiles used to make the predictions may not be indicative of racial/ethnic identity, and (b) it cannot account for Indigenous and mixed-race authors, or those who may face differential biases due to the ambiguous racialization or ethnicization of their names. We look forward to future work that could help us to better understand how to support equitable practices in science.

## Supplementary information

Supplementary Materials

## Data Availability

https://www.ncbi.nlm.nih.gov/projects/gap/cgi-bin/study.cgi?study_id=phs000607.v3.p2.

## Code availability

All analytic code can be found at: https://github.com/lindenmp/normative_neurodev_cs_t1.
