# Supplementary material for: Transdiagnostic dimensions of psychopathology explain individuals’ unique deviations from normative neurodevelopment in brain structure
Source: Transl Psychiatry. 2021 Apr 20;11:232. doi: 10.1038/s41398-021-01342-6 (PMC8058055; doi:10.1038/s41398-021-01342-6)
Supplement: Supplementary file 1 — Supplementary Materials [file 41398_2021_1342_MOESM1_ESM.docx]

**Supplementary Materials:**

**Transdiagnostic dimensions of psychopathology explain individuals’ unique deviations from normative neurodevelopment in brain structure**

Linden Parkes, Tyler M. Moore, Monica E. Calkins, Philip A. Cook, Matthew Cieslak, David R. Roalf, Daniel H. Wolf, Ruben C. Gur, Raquel E. Gur, Theodore D. Satterthwaite, Danielle S. Bassett

**SUPPLEMENTARY METHODS**

*Participants*

From the original 1,601 participants from the Philadelphia Neurodevelopmental Cohort (PNC)^1^, 160 were excluded due to the presence of gross radiological abnormalities distorting brain anatomy or due to medical history that might impact brain function; those with a history of psychiatric illness were retained. Forty-seven more individuals were excluded because they did not pass rigorous manual and automated quality assurance; one more individual was excluded due to corrupted data, totaling 48 excluded. Next, an additional 17 individuals were excluded due to missing demographic data. Finally, 105 more individuals were excluded because they shared a familial link with other individuals in the PNC (most commonly sibling relations). This process left a final sample of 1,271 participants. Note that this is a larger sample than studies of normative brain development that have used the PNC; unlike previous reports, we did not exclude based on history of psychiatric illness. Indeed, previous work has illustrated that this broader coverage of the PNC yields prevalence rates of mental disorders consistent with population norms^2^.

*Imaging data acquisition*

MRI data were acquired on a 3 Tesla Siemens Tim Trio scanner with a 32-channel head coil at the Hospital of the University of Pennsylvania. A 5-min magnetization-prepared, rapid acquisition gradient-echo T1-weighted (MPRAGE) image (TR = 1810ms, TE = 3.51ms, FOV = 180 x 240mm, matrix 256 x 192, voxel resolution of 1mm^3^) was acquired for each participant.

*Imaging data quality control*

All T1-weighted images underwent rigorous quality control by three highly trained image analysts (see Ref. ^3^ for details). Briefly, all images were visually inspected and evaluated for the presence of artifacts. Images with gross artifacts were considered unusable; images with some artifacts were flagged as ‘decent’; and images free of artifact were marked as ‘superior’. Ratings of scan quality were averaged over the three raters and we hereafter refer to this average measure as T1 quality assurance (T1 QA). As mentioned above in the section titled *Participants*, 47 individuals were removed due to unusable imaging data according to this T1 QA measure. As a result, 1,054 (83%) of our 1,271 participants had T1-weighted images identified as ‘superior’, with the remaining identified as ‘usable’.

In addition to the above rater-based estimate of T1-weighted image quality, we also estimated the signal-to-noise ratio of each participant’s T1-weighted image (T1 SNR; see Ref. ^3^ for details) as a secondary measure of image quality.

*Whole brain parcellation*

We summarized cortical volume at the region level. Analyses reported in the main text were conducted using 400 regions covering the cortex that were defined using functional neuroimaging data in a previous study^4^. This set of regions is hereafter referred as the Schaefer400 parcellation. However, there exists a plethora of parcellations in neuroimaging research that vary in their construction. In light of this diversity, we sought to confirm that our results were not driven by choice of brain parcellation, and thus we repeated our analyses using a separate parcellation wherein boundaries were defined according to neuroanatomy rather than function^5^ and that included 463 regions (Lausanne463). For each parcellation, brain features were generated for each participant as described in the following sections.

*Structural image processing*

Structural image processing used tools included in ANTs^6^. Structural images were processed in participant’s native space using the following procedure: brain extraction, N4 bias field correction^7^, Atropos tissue segmentation^8^, and SyN diffeomorphic registration^9,10^. Regional estimates of cortical volume were extracted from every participant’s native space data using the following procedure. First, we created a custom adolescent template and tissue priors using data from 140 PNC participants, balanced for age and sex. A custom template minimizes registration bias and maximizes sensitivity to detect regional effects that can be impacted by registration error. Second, the Schaefer400 parcellation was generated in this template space. Third, nonlinear registration warps were generated that mapped each participant’s structural scan to template space, and the inverse of these warps were applied to the Schaefer400 parcellation to generate participant-specific parcellations. Participant-specific Lausanne463 parcellations were generated in native space data using code available online: <https://github.com/mattcieslak/easy_lausanne>. Fourth, each participant’s Schaefer400 parcellation was masked by a cortical gray matter mask from ANTs, also in participant’s native space. Finally, regional cortical volume estimates were extracted using these participant-specific parcellations (Schafer400, Lausanne463) as the count of the number of voxels in each parcel.

*Regional analysis of a priori regions of interest*

In the main text, we reported analyzing relationships between psychopathology dimensions and deviations in the vmPFC/mOFC, inferior temporal, daCC and insular cortices. In particular, we referenced the fact that these regions were generated by averaging deviations of subsets of Schaefer parcels (see Figure 2). Here, we provide a full list of the names of these parcels.

The vmPFC/mOFC was comprised of the following parcels:

17Networks_LH_Limbic_OFC_1, 17Networks_LH_Limbic_OFC_2, 17Networks_LH_Limbic_OFC_3, 17Networks_LH_Limbic_OFC_4, 17Networks_LH_Limbic_OFC_5, 17Networks_LH_SalVentAttnB_OFC_1, 17Networks_RH_Limbic_OFC_1, 17Networks_RH_Limbic_OFC_2, 17Networks_RH_Limbic_OFC_3, 17Networks_RH_Limbic_OFC_4, 17Networks_RH_Limbic_OFC_5, 17Networks_RH_Limbic_OFC_6.

The inferior temporal cortex was comprised of the following parcels:

17Networks_LH_Limbic_TempPole_1, 17Networks_LH_Limbic_TempPole_2, 17Networks_LH_Limbic_TempPole_3, 17Networks_LH_Limbic_TempPole_4,

17Networks_LH_Limbic_TempPole_5, 17Networks_LH_Limbic_TempPole_6, 17Networks_LH_Limbic_TempPole_7, 17Networks_LH_ContB_Temp_1,

17Networks_LH_DefaultB_Temp_1, 17Networks_LH_DefaultB_Temp_2, 17Networks_LH_DefaultB_Temp_3, 17Networks_LH_DefaultB_Temp_4, 17Networks_LH_DefaultB_Temp_5, 17Networks_LH_DefaultB_Temp_6,

17Networks_RH_Limbic_TempPole_1, 17Networks_RH_Limbic_TempPole_2, 17Networks_RH_Limbic_TempPole_3, 17Networks_RH_Limbic_TempPole_4, 17Networks_RH_Limbic_TempPole_5, 17Networks_RH_Limbic_TempPole_6,

17Networks_RH_ContB_Temp_1, 17Networks_RH_ContB_Temp_2,

17Networks_RH_DefaultA_Temp_1, 17Networks_RH_DefaultB_Temp_1, 17Networks_RH_DefaultB_Temp_2, 17Networks_RH_DefaultB_AntTemp_1.

The daCC was comprised of the following parcels:

17Networks_LH_SalVentAttnB_PFCmp_1, 17Networks_LH_DefaultA_PFCm_6, 17Networks_LH_ContA_Cinga_1, 17Networks_RH_SalVentAttnB_PFCmp_1, 17Networks_RH_SalVentAttnB_PFCmp_2, 17Networks_RH_DefaultA_PFCm_6, 17Networks_RH_ContA_Cinga_1.

The insular cortex was comprised of the following parcels:

17Networks_LH_SalVentAttnA_Ins_1, 17Networks_LH_SalVentAttnA_Ins_2, 17Networks_LH_SalVentAttnA_Ins_3, 17Networks_LH_SalVentAttnA_Ins_4, 17Networks_LH_SalVentAttnA_Ins_5, 17Networks_LH_SalVentAttnA_Ins_6

17Networks_RH_SalVentAttnA_Ins_1, 17Networks_RH_SalVentAttnA_Ins_2, 17Networks_RH_SalVentAttnA_Ins_3, 17Networks_RH_SalVentAttnA_Ins_4,

17Networks_RH_SalVentAttnA_Ins_5, 17Networks_RH_SalVentAttnA_Ins_6, 17Networks_RH_SalVentAttnA_Ins_7.


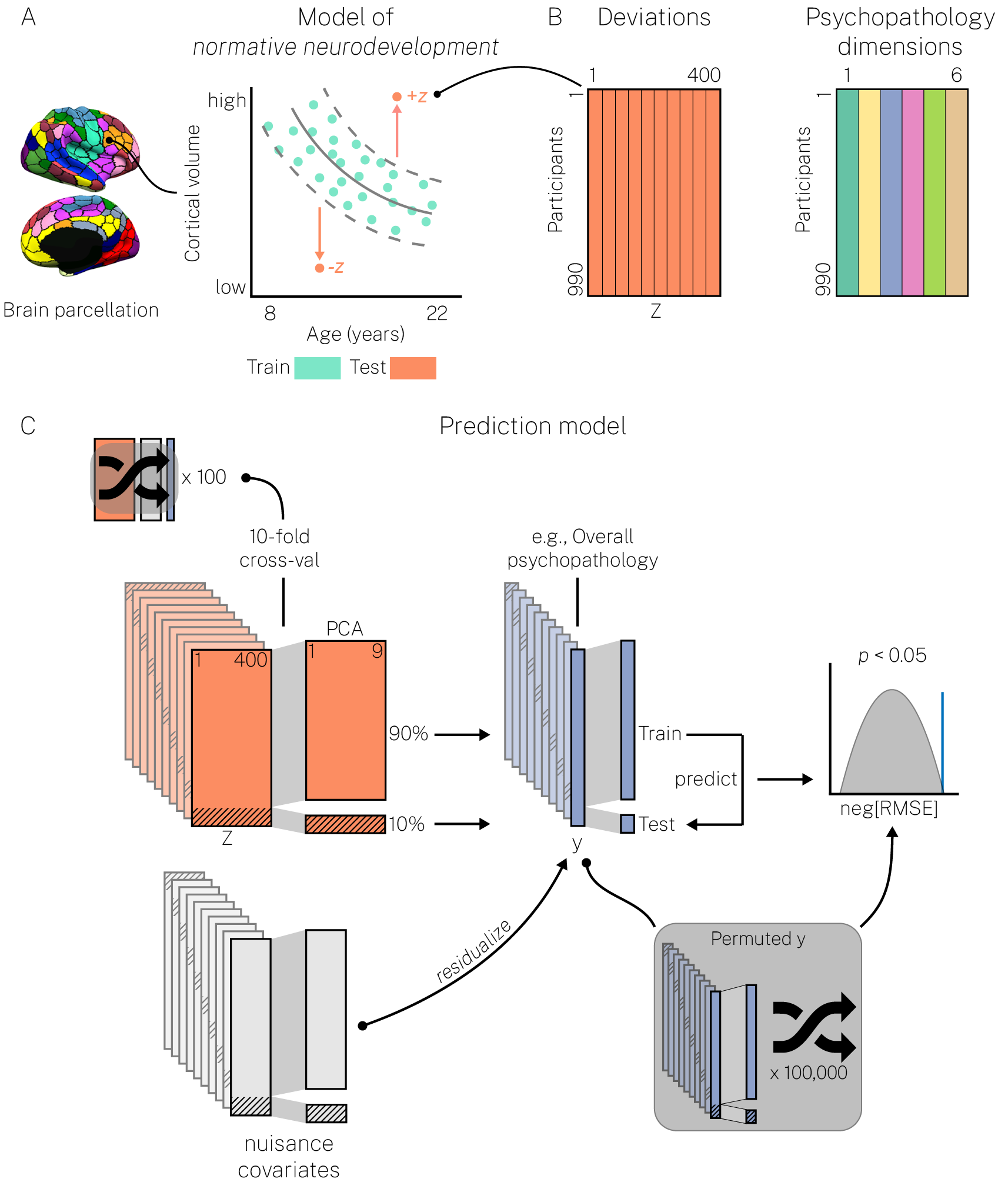


**Figure S1. Machine learning prediction model.** **A**, We extracted cortical volume from 400 regions covering the cortex and estimated a normative model of neurodevelopment in 281 healthy controls (train, ). **B**, Deviations (Z) were modeled in the remaining 990 individuals (test, ) and paired with six dimensions of psychopathology. **C**, Deviations were used to iteratively predict psychopathology dimensions ($y$) using ridge regression and 100 repeats of 10-fold cross-validation. Principal component analysis (PCA) was applied to reduce the deviations to 9 PCs. This procedure was performed done within cross-validation folds to avoid leakage. Additionally, nuisance covariates (e.g., age, sex) were controlled for by adjusting $y$ prior to prediction, also within cross-validation folds to avoid leakage. The ensuing 100 estimates of predictive performance (e.g., negative root mean squared error, neg[RMSE]) were averaged into a single point estimate and significance was assessed against an empirical null generated by randomly permuting $y$ 100,000 times.

**SUPPLEMENTARY RESULTS**

*Dimensional measures of psychopathology*

Below we illustrate model statistics (Table S1) and factor loadings (Table S2) for our bifactor model of psychopathology.

Table S1. Factor determinacy and Omega-H scores for the bifactor model of psychopathology dimensions.

| Item | General  (‘*p*’) | Psychosis-positive | Psychosis-negative | Anxious-misery | Externalizing | Fear |
| --- | --- | --- | --- | --- | --- | --- |
| Factor determinacy | 0.9927 | 0.9600 | 0.9683 | 0.9548 | 0.9661 | 0.9502 |
| Omega-H_subscale_ | 0.9213 | 0.0154 | 0.0056 | 0.0004 | 0.0276 | 0.0192 |

Table S2. Factor loadings from the bifactor model of psychopathology dimensions.

|  | Loadings | | | | | |
| --- | --- | --- | --- | --- | --- | --- |
| Item | General  (‘*p*’) | Psychosis-positive | Psychosis-negative | Anxious-misery | Externalizing | Fear |
| psy001 | **0.657** | **0.442** | 0.000 | 0.000 | 0.000 | 0.000 |
| psy029 | **0.606** | **0.411** | 0.000 | 0.000 | 0.000 | 0.000 |
| psy050 | **0.632** | **0.220** | 0.000 | 0.000 | 0.000 | 0.000 |
| psy060 | **0.666** | **0.316** | 0.000 | 0.000 | 0.000 | 0.000 |
| psy070 | **0.637** | **0.285** | 0.000 | 0.000 | 0.000 | 0.000 |
| psy071 | **0.721** | **0.187** | 0.000 | 0.000 | 0.000 | 0.000 |
| sip003 | **0.598** | **0.522** | 0.000 | 0.000 | 0.000 | 0.000 |
| sip004 | **0.422** | **0.616** | 0.000 | 0.000 | 0.000 | 0.000 |
| sip005 | **0.593** | **0.605** | 0.000 | 0.000 | 0.000 | 0.000 |
| sip006 | **0.557** | **0.559** | 0.000 | 0.000 | 0.000 | 0.000 |
| sip007 | **0.584** | **0.608** | 0.000 | 0.000 | 0.000 | 0.000 |
| sip008 | **0.519** | **0.628** | 0.000 | 0.000 | 0.000 | 0.000 |
| sip009 | **0.615** | **0.502** | 0.000 | 0.000 | 0.000 | 0.000 |
| sip010 | **0.437** | **0.666** | 0.000 | 0.000 | 0.000 | 0.000 |
| sip011 | **0.623** | **0.607** | 0.000 | 0.000 | 0.000 | 0.000 |
| sip012 | **0.639** | **0.596** | 0.000 | 0.000 | 0.000 | 0.000 |
| sip013 | **0.605** | **0.593** | 0.000 | 0.000 | 0.000 | 0.000 |
| sip014 | **0.715** | **0.489** | 0.000 | 0.000 | 0.000 | 0.000 |
| sip027 | **0.487** | 0.000 | **0.288** | 0.000 | 0.000 | 0.000 |
| sip028 | **0.517** | 0.000 | **0.305** | 0.000 | 0.000 | 0.000 |
| sip032 | **0.758** | 0.000 | **0.188** | 0.000 | 0.000 | 0.000 |
| sip033 | **0.681** | 0.000 | **0.205** | 0.000 | 0.000 | 0.000 |
| sip038 | **0.396** | 0.000 | **0.795** | 0.000 | 0.000 | 0.000 |
| sip039 | **0.483** | 0.000 | **0.631** | 0.000 | 0.000 | 0.000 |
| SIP030 | **0.524** | 0.000 | **0.383** | 0.000 | 0.000 | 0.000 |
| SIP035 | **0.714** | 0.000 | **0.302** | 0.000 | 0.000 | 0.000 |
| SIP037 | **0.387** | 0.000 | **0.395** | 0.000 | 0.000 | 0.000 |
| SIP041 | **0.459** | 0.000 | **0.846** | 0.000 | 0.000 | 0.000 |
| SIP043 | **0.496** | 0.000 | **0.678** | 0.000 | 0.000 | 0.000 |
| SIP001 | **0.461** | 0.000 | **0.328** | 0.000 | 0.000 | 0.000 |
| add011 | **0.473** | 0.000 | 0.000 | 0.000 | **0.745** | 0.000 |
| add012 | **0.458** | 0.000 | 0.000 | 0.000 | **0.749** | 0.000 |
| add013 | **0.490** | 0.000 | 0.000 | 0.000 | **0.596** | 0.000 |
| add014 | **0.442** | 0.000 | 0.000 | 0.000 | **0.606** | 0.000 |
| add015 | **0.499** | 0.000 | 0.000 | 0.000 | **0.565** | 0.000 |
| add016 | **0.510** | 0.000 | 0.000 | 0.000 | **0.678** | 0.000 |
| add020 | **0.497** | 0.000 | 0.000 | 0.000 | **0.543** | 0.000 |
| add021 | **0.448** | 0.000 | 0.000 | 0.000 | **0.599** | 0.000 |
| add022 | **0.468** | 0.000 | 0.000 | 0.000 | **0.603** | 0.000 |
| agr001 | **0.611** | 0.000 | 0.000 | 0.000 | 0.000 | **0.474** |
| agr002 | **0.635** | 0.000 | 0.000 | 0.000 | 0.000 | **0.489** |
| agr003 | **0.651** | 0.000 | 0.000 | 0.000 | 0.000 | **0.421** |
| agr004 | **0.550** | 0.000 | 0.000 | 0.000 | 0.000 | **0.422** |
| agr005 | **0.523** | 0.000 | 0.000 | 0.000 | 0.000 | **0.469** |
| agr006 | **0.620** | 0.000 | 0.000 | 0.000 | 0.000 | **0.457** |
| agr007 | **0.621** | 0.000 | 0.000 | 0.000 | 0.000 | **0.286** |
| agr008 | **0.621** | 0.000 | 0.000 | 0.000 | 0.000 | **0.453** |
| cdd001 | **0.573** | 0.000 | 0.000 | 0.000 | **0.407** | 0.000 |
| cdd002 | **0.548** | 0.000 | 0.000 | 0.000 | **0.219** | 0.000 |
| cdd003 | **0.621** | 0.000 | 0.000 | 0.000 | **0.462** | 0.000 |
| cdd004 | **0.468** | 0.000 | 0.000 | 0.000 | **0.334** | 0.000 |
| cdd005 | **0.606** | 0.000 | 0.000 | 0.000 | **0.477** | 0.000 |
| cdd006 | **0.613** | 0.000 | 0.000 | 0.000 | **0.384** | 0.000 |
| cdd007 | **0.635** | 0.000 | 0.000 | 0.000 | **0.372** | 0.000 |
| cdd008 | **0.637** | 0.000 | 0.000 | 0.000 | **0.348** | 0.000 |
| dep001 | **0.760** | 0.000 | 0.000 | **0.220** | 0.000 | 0.000 |
| dep002 | **0.724** | 0.000 | 0.000 | **0.187** | 0.000 | 0.000 |
| dep004 | **0.791** | 0.000 | 0.000 | **0.031** | 0.000 | 0.000 |
| dep006 | **0.775** | 0.000 | 0.000 | **0.034** | 0.000 | 0.000 |
| gad001 | **0.506** | 0.000 | 0.000 | **0.377** | 0.000 | 0.000 |
| gad002 | **0.554** | 0.000 | 0.000 | **0.404** | 0.000 | 0.000 |
| man001 | **0.743** | 0.000 | 0.000 | **-0.517** | 0.000 | 0.000 |
| man002 | **0.744** | 0.000 | 0.000 | **-0.567** | 0.000 | 0.000 |
| man003 | **0.732** | 0.000 | 0.000 | **-0.523** | 0.000 | 0.000 |
| man004 | **0.771** | 0.000 | 0.000 | **-0.456** | 0.000 | 0.000 |
| man005 | **0.767** | 0.000 | 0.000 | **-0.460** | 0.000 | 0.000 |
| man006 | **0.689** | 0.000 | 0.000 | **-0.487** | 0.000 | 0.000 |
| man007 | **0.808** | 0.000 | 0.000 | **-0.241** | 0.000 | 0.000 |
| ocd001 | **0.844** | 0.000 | 0.000 | **0.197** | 0.000 | 0.000 |
| ocd002 | **0.807** | 0.000 | 0.000 | **0.125** | 0.000 | 0.000 |
| ocd003 | **0.709** | 0.000 | 0.000 | **0.209** | 0.000 | 0.000 |
| ocd004 | **0.826** | 0.000 | 0.000 | **0.060** | 0.000 | 0.000 |
| ocd005 | **0.822** | 0.000 | 0.000 | **0.115** | 0.000 | 0.000 |
| ocd006 | **0.843** | 0.000 | 0.000 | **0.107** | 0.000 | 0.000 |
| ocd007 | **0.665** | 0.000 | 0.000 | **0.143** | 0.000 | 0.000 |
| ocd008 | **0.766** | 0.000 | 0.000 | **0.131** | 0.000 | 0.000 |
| ocd011 | **0.712** | 0.000 | 0.000 | **0.196** | 0.000 | 0.000 |
| ocd012 | **0.721** | 0.000 | 0.000 | **0.134** | 0.000 | 0.000 |
| ocd013 | **0.699** | 0.000 | 0.000 | **0.119** | 0.000 | 0.000 |
| ocd014 | **0.763** | 0.000 | 0.000 | **0.061** | 0.000 | 0.000 |
| ocd015 | **0.732** | 0.000 | 0.000 | **0.092** | 0.000 | 0.000 |
| ocd016 | **0.714** | 0.000 | 0.000 | **0.150** | 0.000 | 0.000 |
| ocd017 | **0.719** | 0.000 | 0.000 | **0.090** | 0.000 | 0.000 |
| ocd018 | **0.629** | 0.000 | 0.000 | **0.095** | 0.000 | 0.000 |
| ocd019 | **0.561** | 0.000 | 0.000 | **0.073** | 0.000 | 0.000 |
| odd001 | **0.588** | 0.000 | 0.000 | 0.000 | **0.436** | 0.000 |
| odd002 | **0.573** | 0.000 | 0.000 | 0.000 | **0.515** | 0.000 |
| odd003 | **0.532** | 0.000 | 0.000 | 0.000 | **0.568** | 0.000 |
| odd005 | **0.553** | 0.000 | 0.000 | 0.000 | **0.486** | 0.000 |
| odd006 | **0.634** | 0.000 | 0.000 | 0.000 | **0.397** | 0.000 |
| pan001 | **0.621** | 0.000 | 0.000 | **0.275** | 0.000 | 0.000 |
| pan003 | **0.692** | 0.000 | 0.000 | **0.156** | 0.000 | 0.000 |
| pan004 | **0.779** | 0.000 | 0.000 | **0.159** | 0.000 | 0.000 |
| phb001 | **0.276** | 0.000 | 0.000 | 0.000 | 0.000 | **0.309** |
| phb002 | **0.340** | 0.000 | 0.000 | 0.000 | 0.000 | **0.350** |
| phb003 | **0.422** | 0.000 | 0.000 | 0.000 | 0.000 | **0.282** |
| phb004 | **0.270** | 0.000 | 0.000 | 0.000 | 0.000 | **0.355** |
| phb005 | **0.186** | 0.000 | 0.000 | 0.000 | 0.000 | **0.263** |
| phb006 | **0.456** | 0.000 | 0.000 | 0.000 | 0.000 | **0.314** |
| phb007 | **0.418** | 0.000 | 0.000 | 0.000 | 0.000 | **0.388** |
| phb008 | **0.365** | 0.000 | 0.000 | 0.000 | 0.000 | **0.199** |
| scr001 | **0.494** | 0.000 | 0.000 | 0.000 | **0.163** | 0.000 |
| scr006 | **0.487** | 0.000 | 0.000 | 0.000 | **0.357** | 0.000 |
| scr007 | **0.651** | 0.000 | 0.000 | **0.210** | 0.000 | 0.000 |
| scr008 | **0.545** | 0.000 | 0.000 | 0.000 | **0.255** | 0.000 |
| sep500 | **0.462** | 0.000 | 0.000 | 0.000 | 0.000 | **0.168** |
| sep508 | **0.413** | 0.000 | 0.000 | 0.000 | 0.000 | **0.202** |
| sep509 | **0.433** | 0.000 | 0.000 | 0.000 | 0.000 | **0.226** |
| sep510 | **0.525** | 0.000 | 0.000 | **0.085** | 0.000 | 0.000 |
| sep511 | **0.310** | 0.000 | 0.000 | 0.000 | 0.000 | **0.108** |
| soc001 | **0.444** | 0.000 | 0.000 | 0.000 | 0.000 | **0.638** |
| soc002 | **0.436** | 0.000 | 0.000 | 0.000 | 0.000 | **0.557** |
| soc003 | **0.383** | 0.000 | 0.000 | 0.000 | 0.000 | **0.708** |
| soc004 | **0.449** | 0.000 | 0.000 | 0.000 | 0.000 | **0.685** |
| soc005 | **0.486** | 0.000 | 0.000 | 0.000 | 0.000 | **0.661** |
| sui001 | **0.647** | 0.000 | 0.000 | **0.185** | 0.000 | 0.000 |
| sui002 | **0.740** | 0.000 | 0.000 | **0.260** | 0.000 | 0.000 |

Figure S2 illustrates the average scores on our psychopathology dimensions as a function of the clinical groups present in our sample.


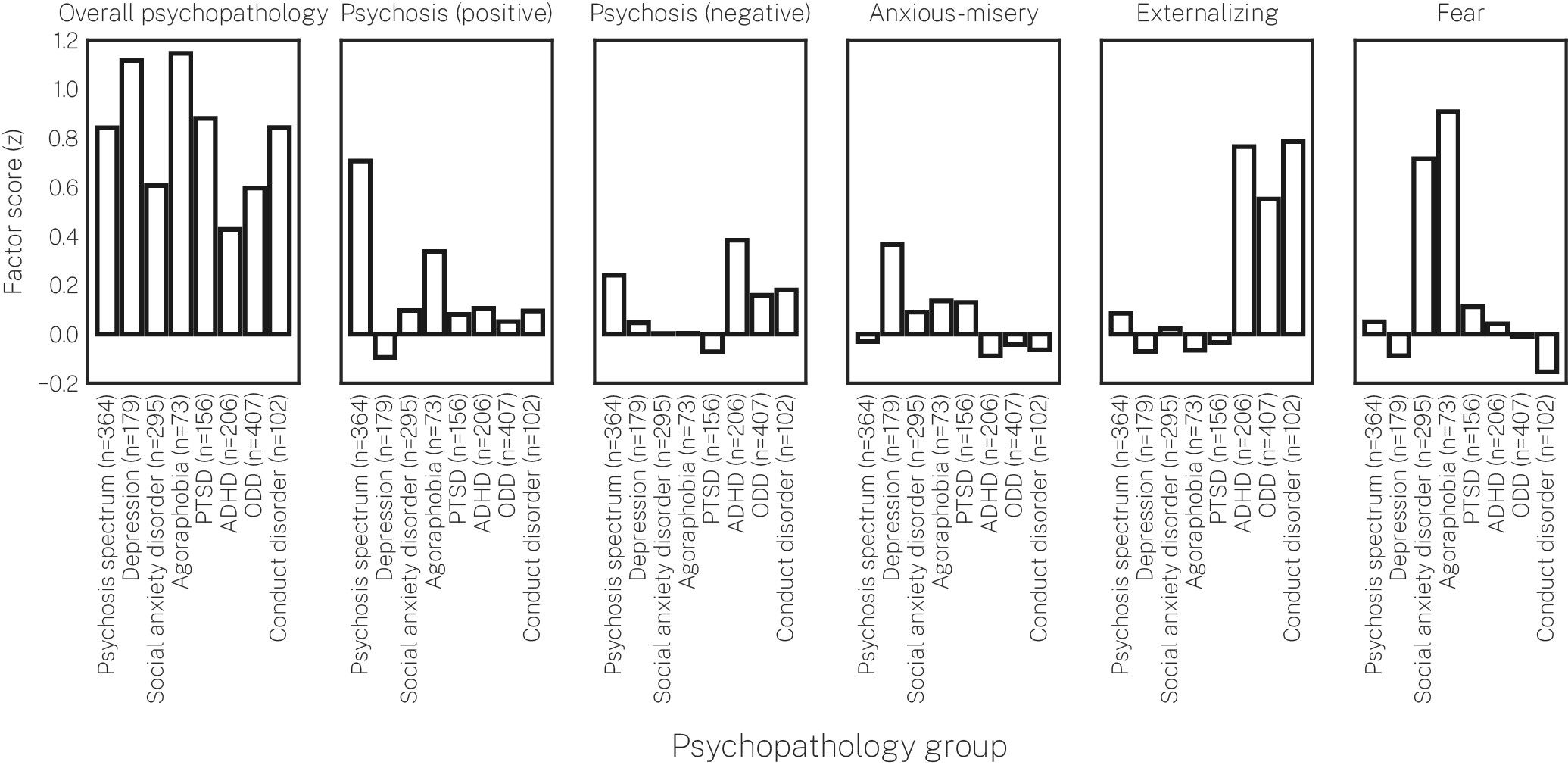


**Figure S2. Mean psychopathology symptom dimensions as a function of psychopathology groups.** Groups are the same as those presented in Table 1 in the main text. Only groups where *n*≥50 are shown here.

Figures S3 and S4 illustrate the effects of sex and age on our psychopathology dimensions. As we often observed clear effects of age and sex, we controlled age and sex in our prediction models (see main text).


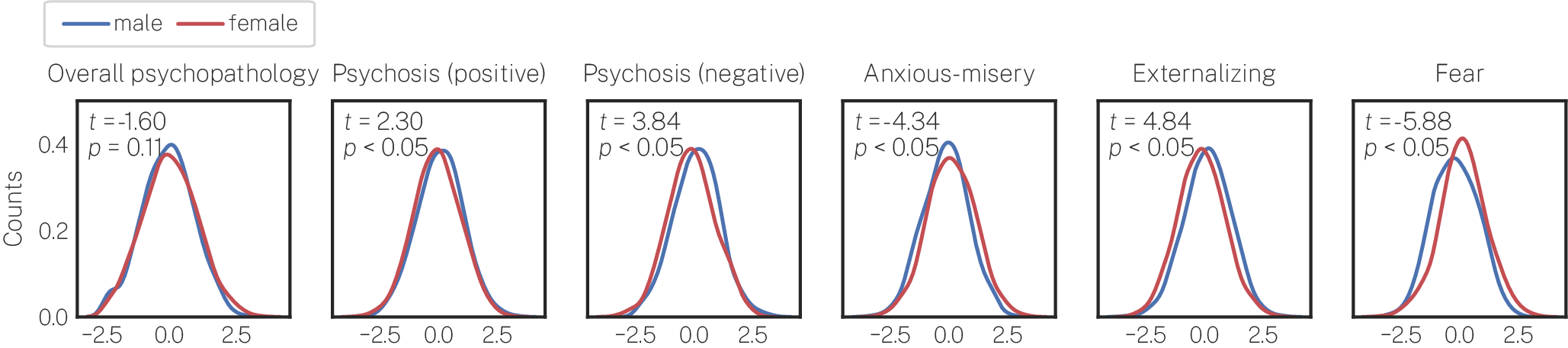


**Figure S3. Sex effects across psychopathology dimensions in the full sample (n=1,271).**


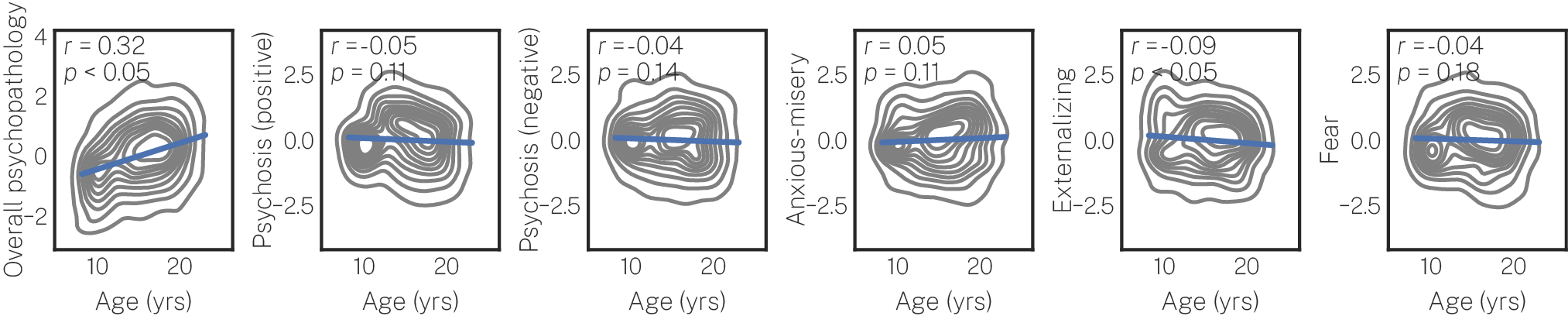


**Figure S4. Age effects across psychopathology dimensions in the full sample (n=1,271).**

*Normative models of cortical volume*

For both males and females, the normative model revealed that greater age was associated with whole brain decreases in cortical volume (Figure S5A). Figure S5B shows the out-of-sample prediction error and explained variance from the normative models run for each brain region. These diagnostics demonstrate that our normative models captured the effects of age well (as a function of sex) across brain regions.


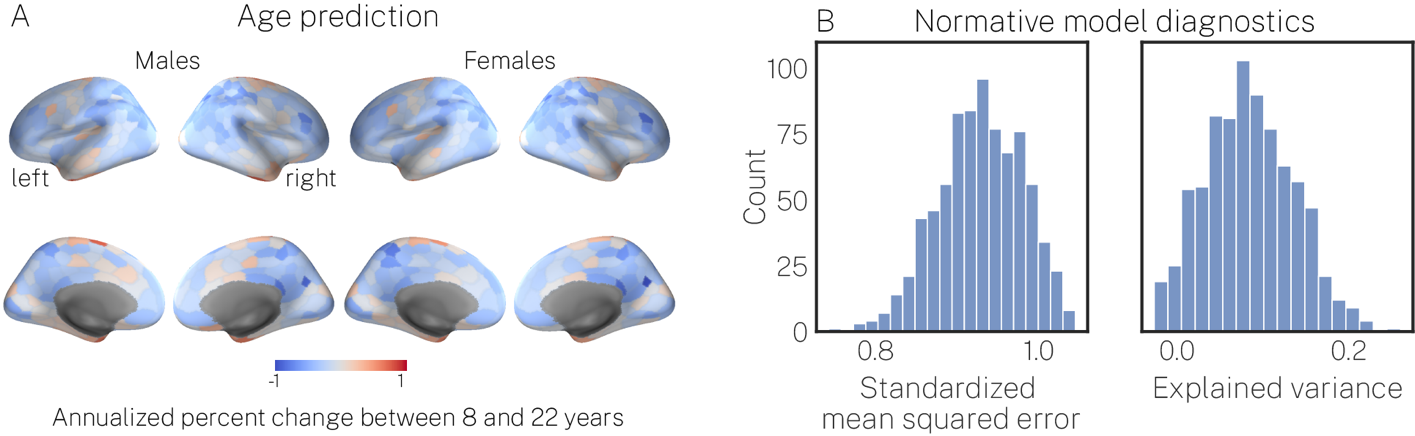


**Figure S5. Age trajectories and model statistics from the normative model.** **A**, Age trajectories for each sex learned by the normative model. **B**, Out-of-sample standardized mean squared error and explained variance from the normative models run for each region in the Schaefer400 parcellation.

In addition to the above, in the normative sample we found that 15.5% of the Schaefer400 regions (62 regions) showed significant Pearson’s correlations to IQ —which we estimated using the Wide Range Achievement Test (WRAT-4)^11^ — and 0.25% (1 region) showed significant Pearson’s correlations to our rater-based estimate of scan quality, T1 QA, and T1 SNR (see section titled *Imaging data quality control* above). This pattern of findings suggests that T1 data quality was unlikely to impact the estimation of our normative models. See section entitled *Sensitivity analyses* below for treatment of IQ in subsequent analyses. Finally, we note that there were no group differences between the train and test subsets on IQ, T1 QA, or T1 SNR (all p-values > 0.05).

*Principal component analysis of deviations from normative neurodevelopment*

In the main text, prior to running our ridge regression prediction models, we reduced each of our multivariate patterns of deviations and raw cortical volume down to their respective dominant sources of variance using principal component analysis (PCA). Figure S6 shows that for both deviations and raw cortical volume estimates, variance in the data was mostly captured by the first PC. Subsequent PCs explained approximately 1% variance each. PCs beyond PC 9 for deviations and PC 8 for raw volume explained <1% variance each. In our regression models (see main text), we sought to retain only PCs that explained at least 1% of the variance. However, in order to maintain an equivalent number of features in our regression models, we retained 9 PCs for both deviations and raw volume. Thus, we retained 1 additional PC for raw volume that had <1% explained variance. The PC coefficients from the first 9 PCs for deviations and raw cortical volume are shown in Figure S7 and Figure S8, respectively.


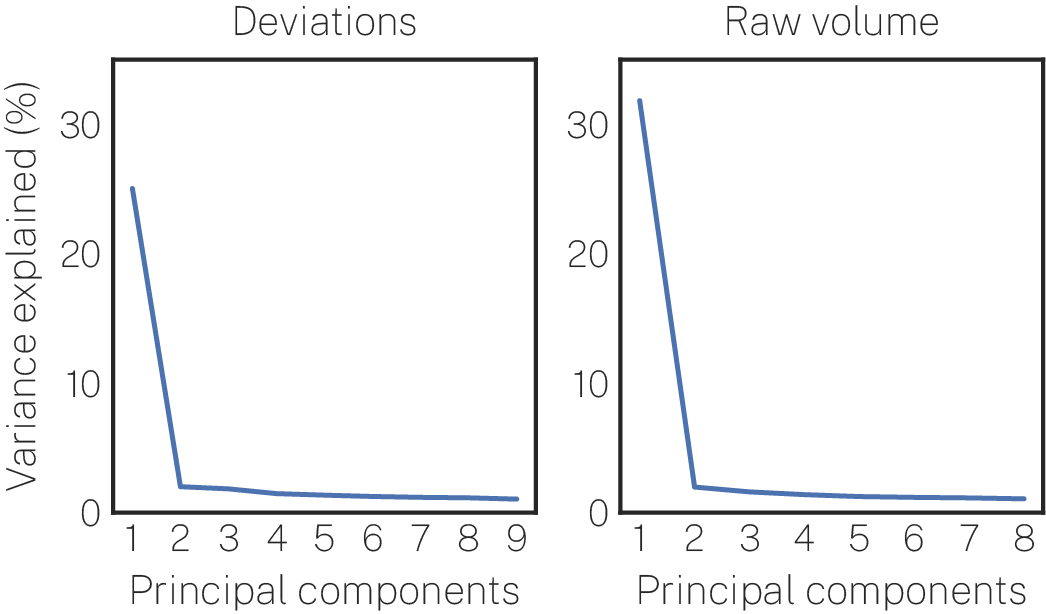


**Figure S6. Variance explained in principal components derived from deviations and raw cortical volume.**


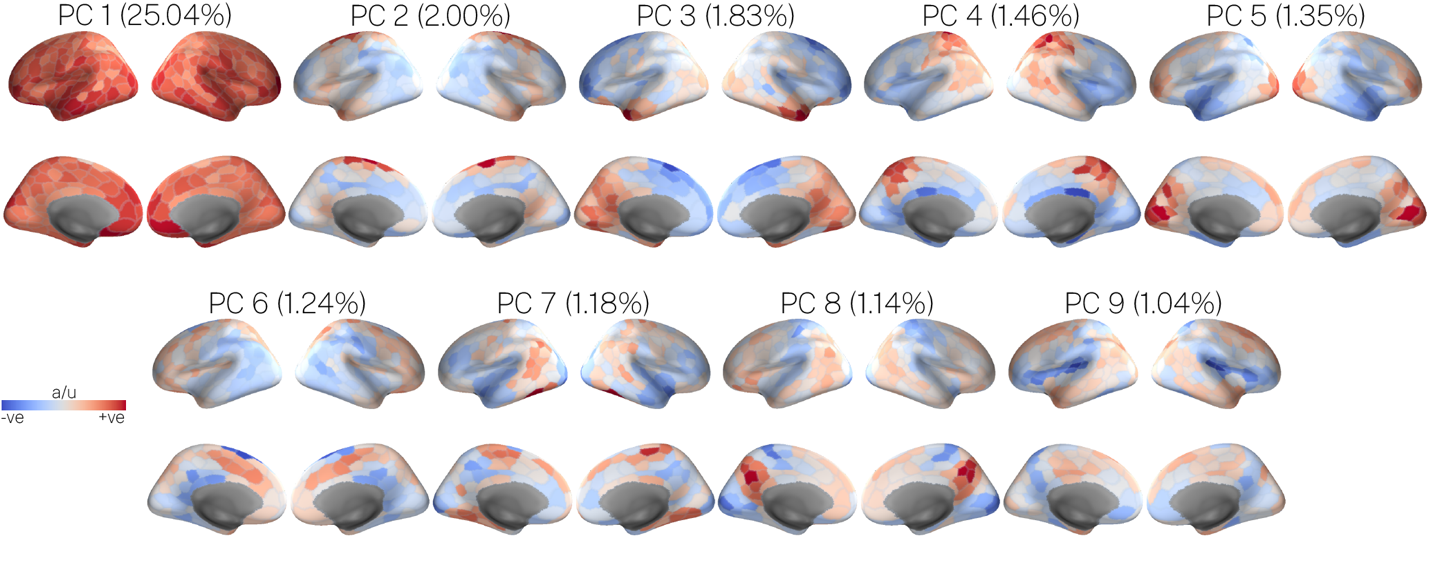


**Figure S7. Coefficients of the principal components derived from deviations from normative neurodevelopment of cortical volume.** The explained variance of each principal component is shown in parentheses.

**
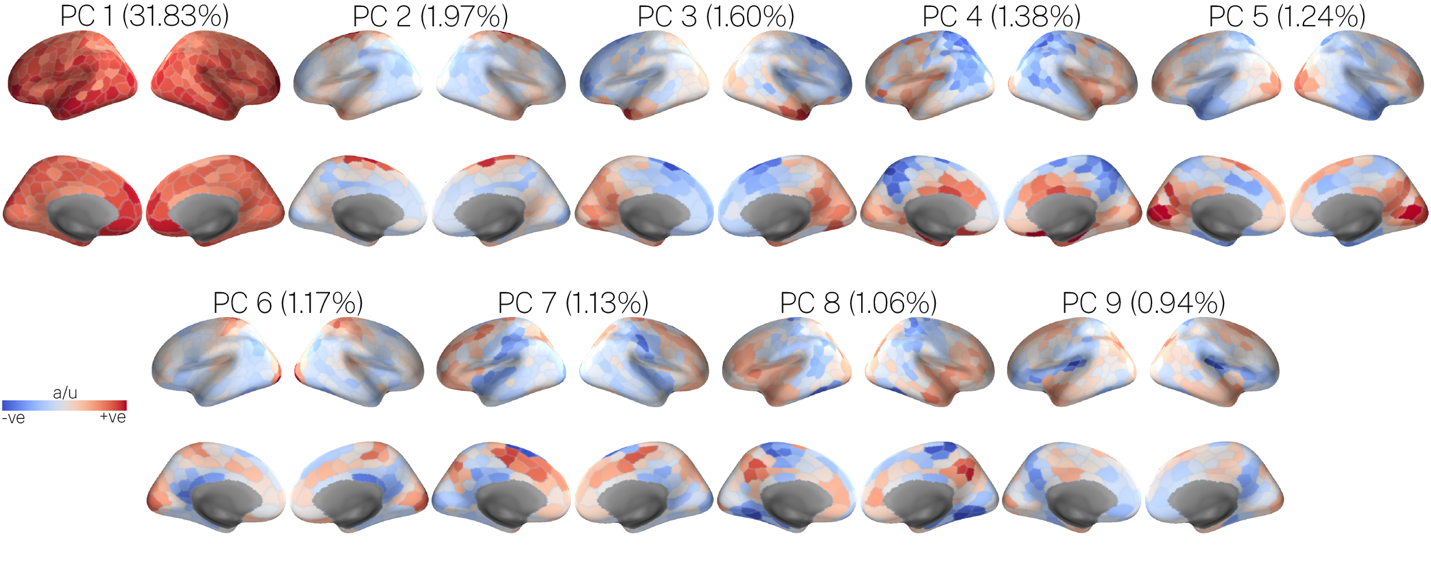
**

**Figure S8.** **Coefficients of the principal components derived from raw cortical volume.** The explained variance of each principal component is shown in parentheses.

*Prediction from individual principal components*

In the main text, we used the above 9 PCs derived from deviations (Figure S7) as well the above 9 PCs derived from raw cortical volume (Figure S8) to predict psychopathology dimensions. Here, to assess the contribution of single PCs to prediction, we repeated our ridge regression prediction 18 times, each time retraining on scores from a single PC taken from either our deviation or raw volume PCs to predict overall psychopathology. Figure S9 shows predictive performance for pairs of PCs taken across deviations and raw cortical volume (e.g., PC 1 from deviations compared to PC 1 from raw cortical volume). We found that only PC 1 from deviations predicted overall psychopathology better than chance levels. Further, PC 1 from deviations yielded significantly better prediction than PC 1 from raw cortical volume, which in itself did not predict beyond chance levels. Thus, the main predictive signal from our PCA of deviations and raw cortical volume came from PC 1.


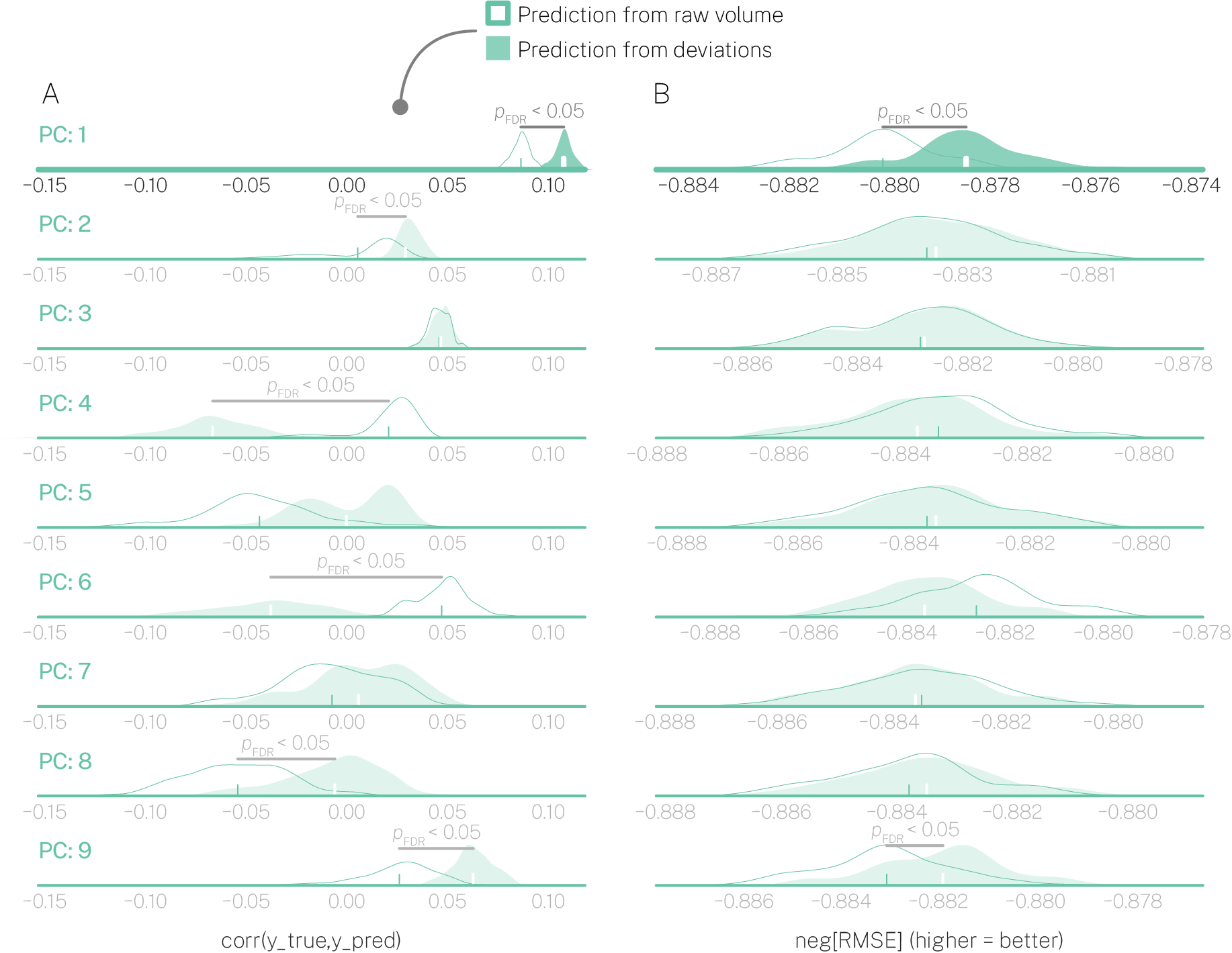


**Figure S9.** **Using individual principal components of variance in raw cortical volume and deviations from normative neurodevelopment to predict overall psychopathology.** Predictive performance for overall psychopathology was assessed separately for each of 9 principal components (PCs) derived from raw cortical volume (Figure S8) and each of 9 PCs derived from deviations from normative neurodevelopment (Figure S7). Specifically, our ridge regression model was retrained using scores from one PC at a time (deviations or raw cortical volume). Separate empirical nulls were generated for each PC. We find that only PC 1 for deviations yielded significant predictive performance. Further, predictive performance for PC 1 from deviations was significantly greater than predictive performance of PC 1 from raw cortical volume, which was in itself not significant.

*Psychoactive medication*

In the main text, our prediction models included n=142 individuals who were taking psychoactive medication. Here, we repeat our prediction model excluding these 142 individuals (Figure S10). Excluding these individuals revealed more significant predictive effects compared to our primary analysis (see Figure 1). In particular, both raw volume and deviations were able to predict psychosis-negative scores above chance levels and deviations were able to predict externalizing scores above chance levels. In these instances, deviations significantly outperformed raw cortical volume. These results suggest that the presence of individuals on psychoactive medications may have confounded our prediction analysis, weakening certain effects. We note that these differences in results could also reflect a change in statistical power, as removing 142 individuals from the test set changed our sample size from n=990 to n=848. Critically, removing these individuals did not diminish the effects we observed in our primary analysis for overall psychopathology, psychosis-positive, and fear, supporting their robustness.


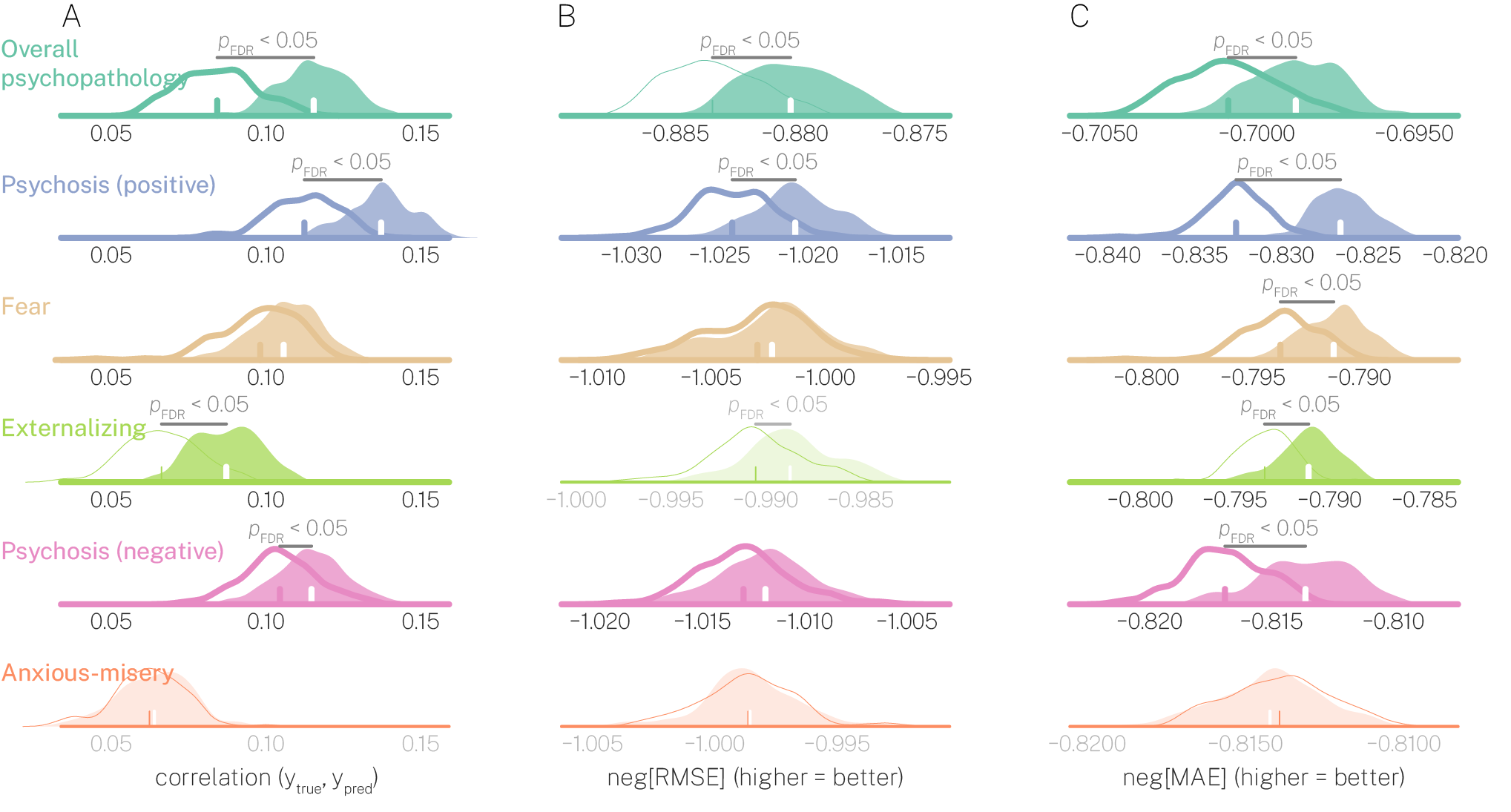


**Figure S10.** **Prediction model excluding n=142 individuals who were on psychoactive medication.** Predictive performance for each of six dimensions of psychopathology (rows) as a function of multiple scoring metrics (columns A-C). In each subplot two distributions are presented: one that illustrates predictive performance derived from raw cortical volume (white distribution with colored outline), and one that illustrates predictive performance derived from deviations from normative models (colored distribution). Distributions of predictive performance that did not yield above chance performance are shown with partial transparency and lighter stroke. Here, nuisance covariates were age and sex.

*Additional nuisance covariates*

In the main text, we reported results for ridge regression prediction models that included age and sex as nuisance covariates (see Figures S3 and S4 above for sex and age effects on psychopathology dimensions). Here, we illustrate the effects of several other potential confounding variables: (i) socioeconomic status, indexed by years of maternal education; (ii) T1 scan quality, indexed by manual T1 QA and T1 SNR (see section above entitled *Imaging data quality control*); and (iii) general intelligence, indexed by the WRAT-4^11^ (see section above entitled *Normative models of cortical volume*). We observed that years of maternal education and general intelligence were significantly correlated with each of our psychopathology dimensions while T1 QA and T1 SNR were not (Figure S11). The lack of significant correlations with T1 QA and T1 SNR may simply reflect the fact that low quality T1 data had already been excluded by this point in our analysis pipeline (see section above entitled *Imaging data quality control*). Nevertheless, for completeness, we performed sensitivity analyses by adding years of maternal education (Figure S12), T1 QA (Figure S13), T1 SNR (Figure S14), and WRAT-4 (Figure S15) as additional covariates alongside age and sex.

Figures S12-S15 show that the effect of deviations significantly outperforming raw cortical volume was largely preserved under our aforementioned sensitivity analyses. However, including years of maternal education (Figure S12) and WRAT-4 (Figure S15) as nuisance covariates resulted in prediction performance for both deviations and raw volume largely dropping below significance for overall psychopathology and fear. Above-chance predictive performance was retained for the psychosis-positive dimension. This pattern of findings suggests that these nuisance covariates may to some degree have confounded our prediction of overall psychopathology and fear, raising the possibility of mediating effects of socioeconomic status and general intelligence on the relationship between brain structure and some measures of psychopathology. Critically, given that deviations remained significantly better predictors compared to raw volume, such a mediation analysis would still benefit from the use of normative models. By contrast, significantly improved predictive performance for deviations relative to raw cortical volume and above-chance predictive performance were both widely retained when using T1 QA and T1 SNR as additional nuisance covariates (Figure S13 and S14).

**
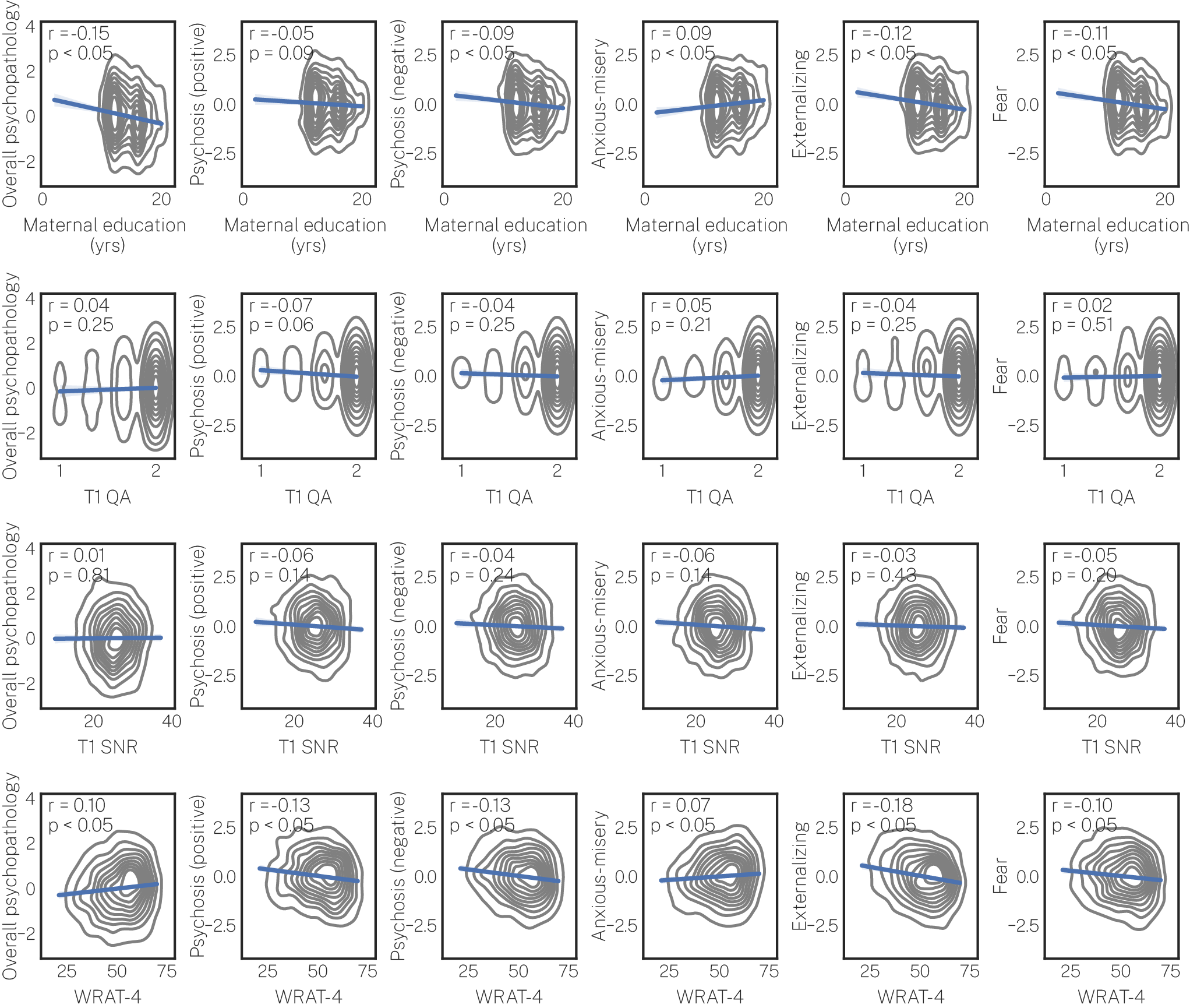
**

**Figure S11. Effect of years of maternal education, T1 QA, T1 SNR, and general intelligence (WRAT-4) on psychopathology dimensions in the full sample (n=1,271).**


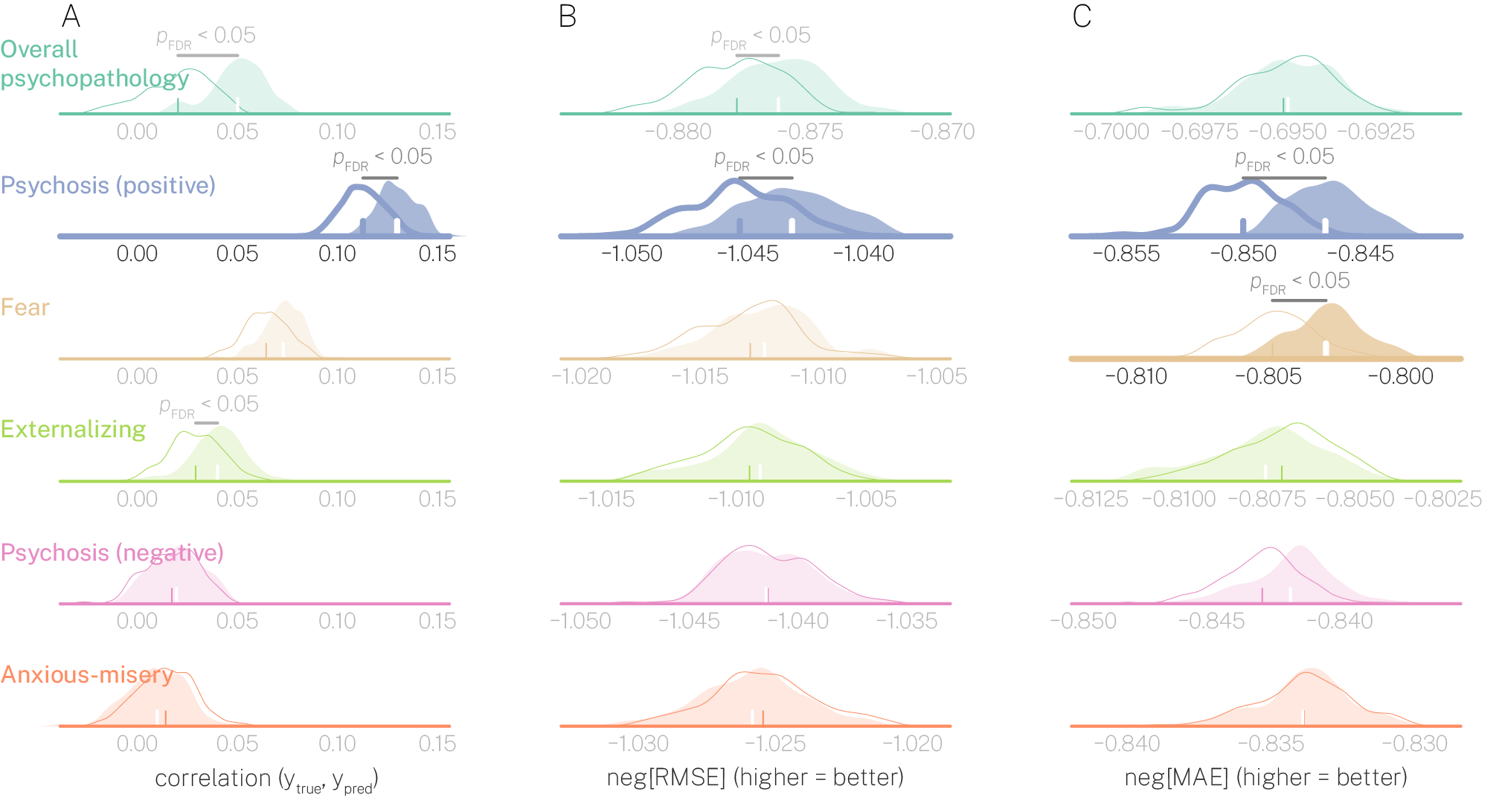


**Figure S12.** **Prediction model including years of maternal education as a nuisance covariate.** Predictive performance for each of six dimensions of psychopathology (rows) as a function of multiple scoring metrics (columns A-C). In each subplot two distributions are presented: one that illustrates predictive performance derived from raw cortical volume (white distribution with colored outline), and one that illustrates predictive performance derived from deviations from normative models (colored distribution). Distributions of predictive performance that did not yield above chance performance are shown with partial transparency and lighter stroke.


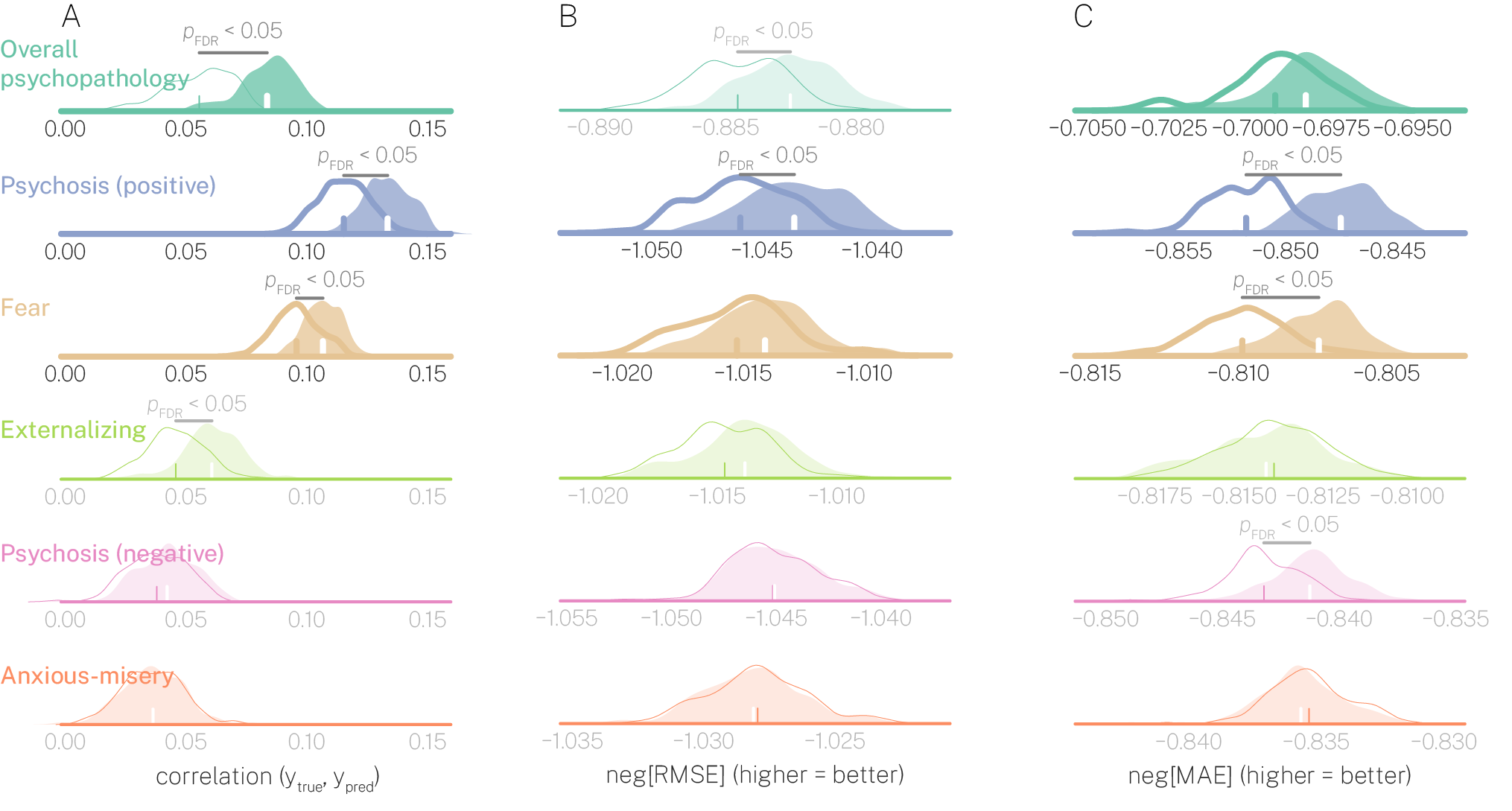


**Figure S13.** **Prediction model including T1 QA as a nuisance covariate.** Predictive performance for each of six dimensions of psychopathology (rows) as a function of multiple scoring metrics (columns A-C). In each subplot two distributions are presented: one that illustrates predictive performance derived from raw cortical volume (white distribution with colored outline), and one that illustrates predictive performance derived from deviations from normative models (colored distribution). Distributions of predictive performance that did not yield above chance performance are shown with partial transparency and lighter stroke.


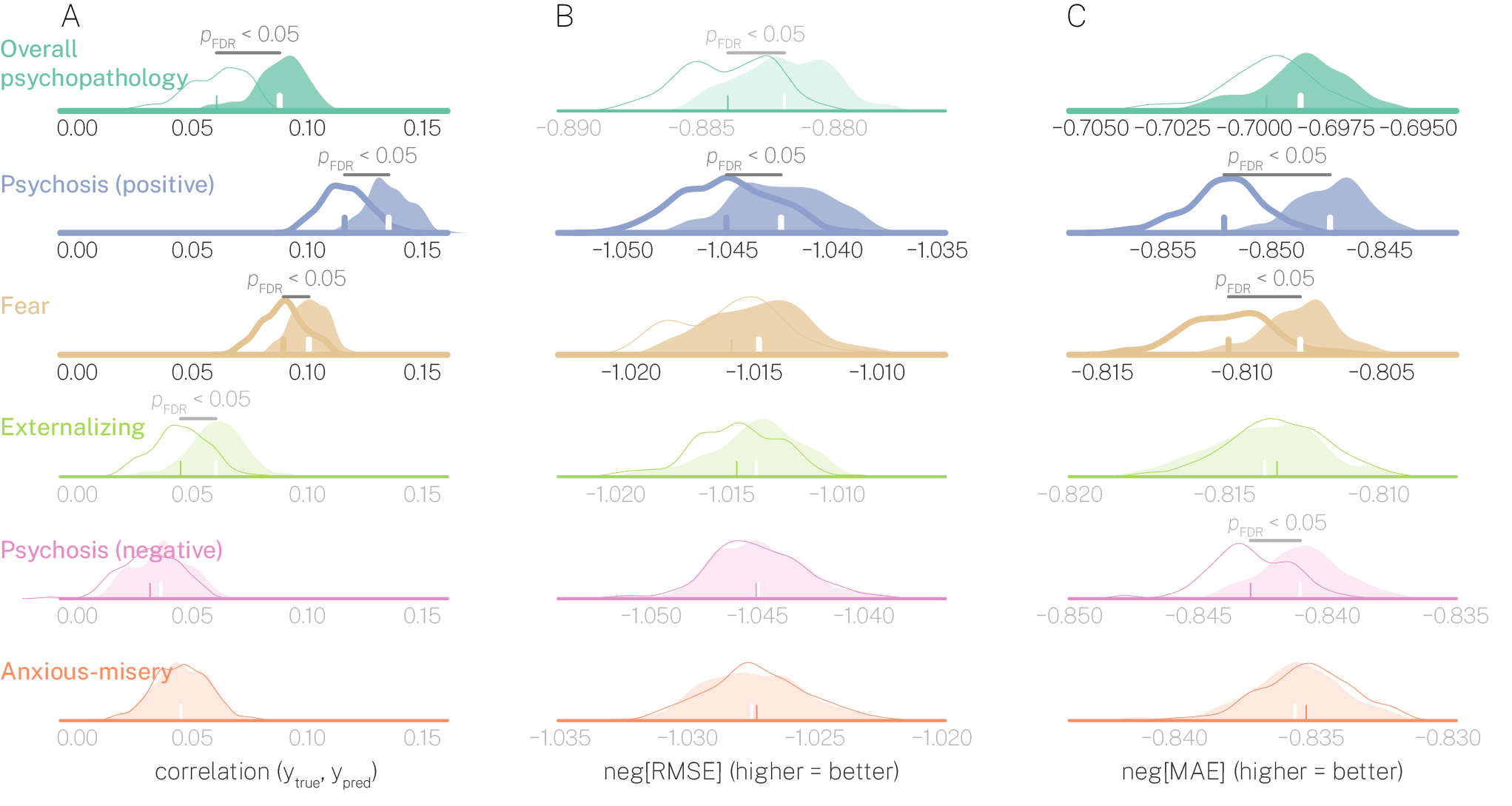


**Figure S14.** **Prediction model including T1 SNR as a nuisance covariate.** Predictive performance for each of six dimensions of psychopathology (rows) as a function of multiple scoring metrics (columns A-C). In each subplot two distributions are presented: one that illustrates predictive performance derived from raw cortical volume (white distribution with colored outline), and one that illustrates predictive performance derived from deviations from normative models (colored distribution). Distributions of predictive performance that did not yield above chance performance are shown with partial transparency and lighter stroke.

**
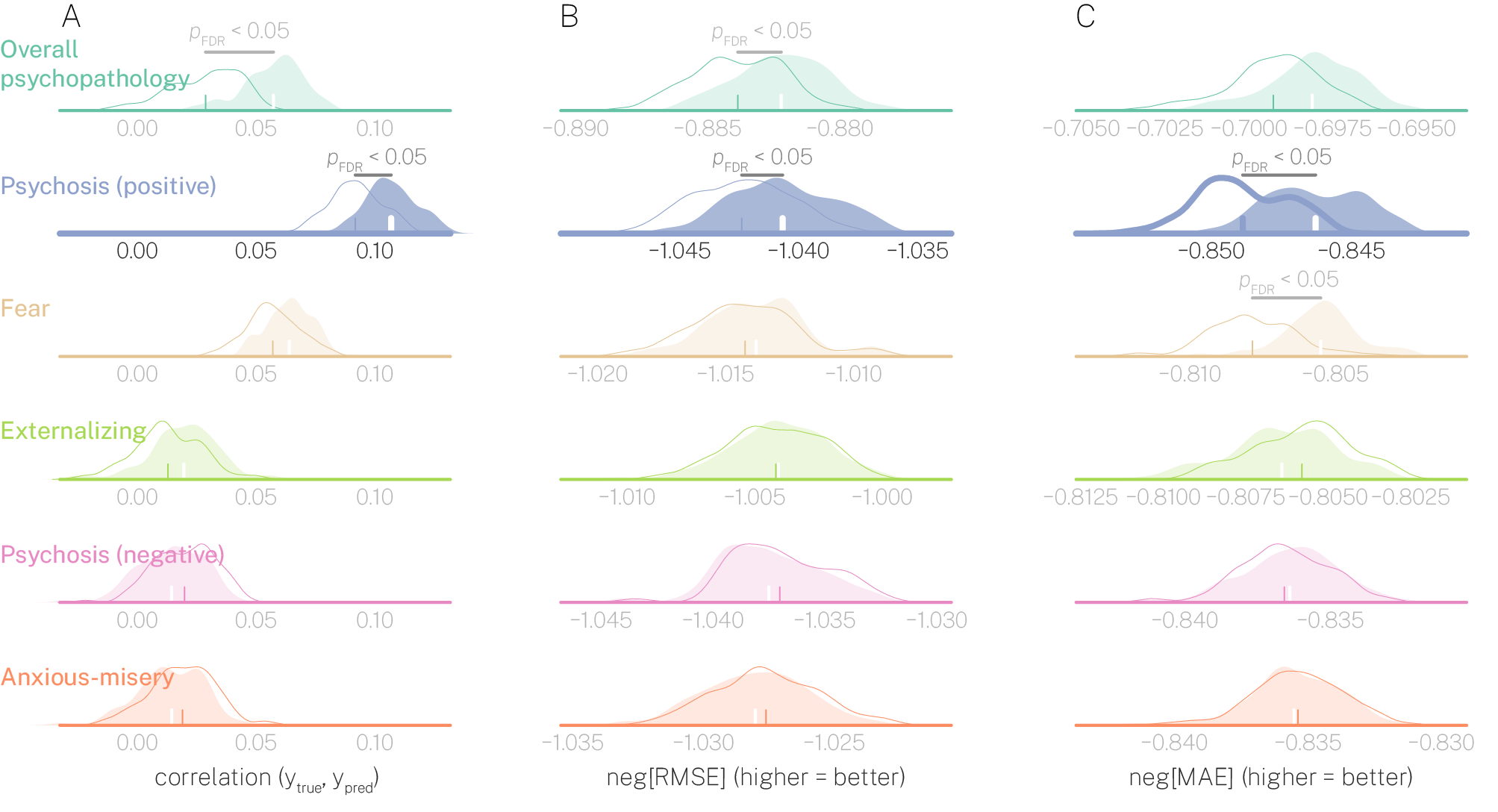
**

**Figure S15.** **Prediction model including general intelligence as a nuisance covariate.** Predictive performance for each of six dimensions of psychopathology (rows) as a function of multiple scoring metrics (columns A-C). In each subplot two distributions are presented: one that illustrates predictive performance derived from raw cortical volume (white distribution with colored outline), and one that illustrates predictive performance derived from deviations from normative models (colored distribution). Distributions of predictive performance that did not yield above chance performance are shown with partial transparency and lighter stroke.

*Parcellation scheme*

In the main text, we reported results for the Schaefer400 parcellation. Here, we repeated our ridge regression prediction model, scored using the correlations between true $y$ and predicted $y$, for a different parcellation known as the Lausanne atlas with 463 regions. Consistent with our primary results, when controlling for age and sex, we found that deviations yielded significantly better prediction performance for the overall psychopathology (raw volume, mean *r* = 0.070; deviations, mean *r* = 0.086; *p* < 0.05_FDR_), psychosis-positive (raw volume, mean *r* = 0.010; deviations, mean *r* = 0.028; *p* < 0.05_FDR_), and fear dimensions (raw volume, mean *r* = 0.052; deviations, mean *r* = 0.069; *p* < 0.05_FDR_).

*Psychopathology dimensions explain regional deviations from normative neurodevelopment*

We compared the effect sizes of correlations between overall psychopathology and regional deviations against the effect sizes observed for specific psychopathology dimensions using bootstrapped samples. In the main text, we determined overall psychopathology to have yielded significantly larger effect sizes if the lower bound of the 99% confident interval (CI) of the bootstrapped effect size differences was greater than 0. Here, we repeated that analysis but determined the effect size differences to be significant if the lower bound of the 95% CI was greater than 0 (equivalent to *p* < 0.05, uncorrected). These results are shown in Figure S16 below.

**
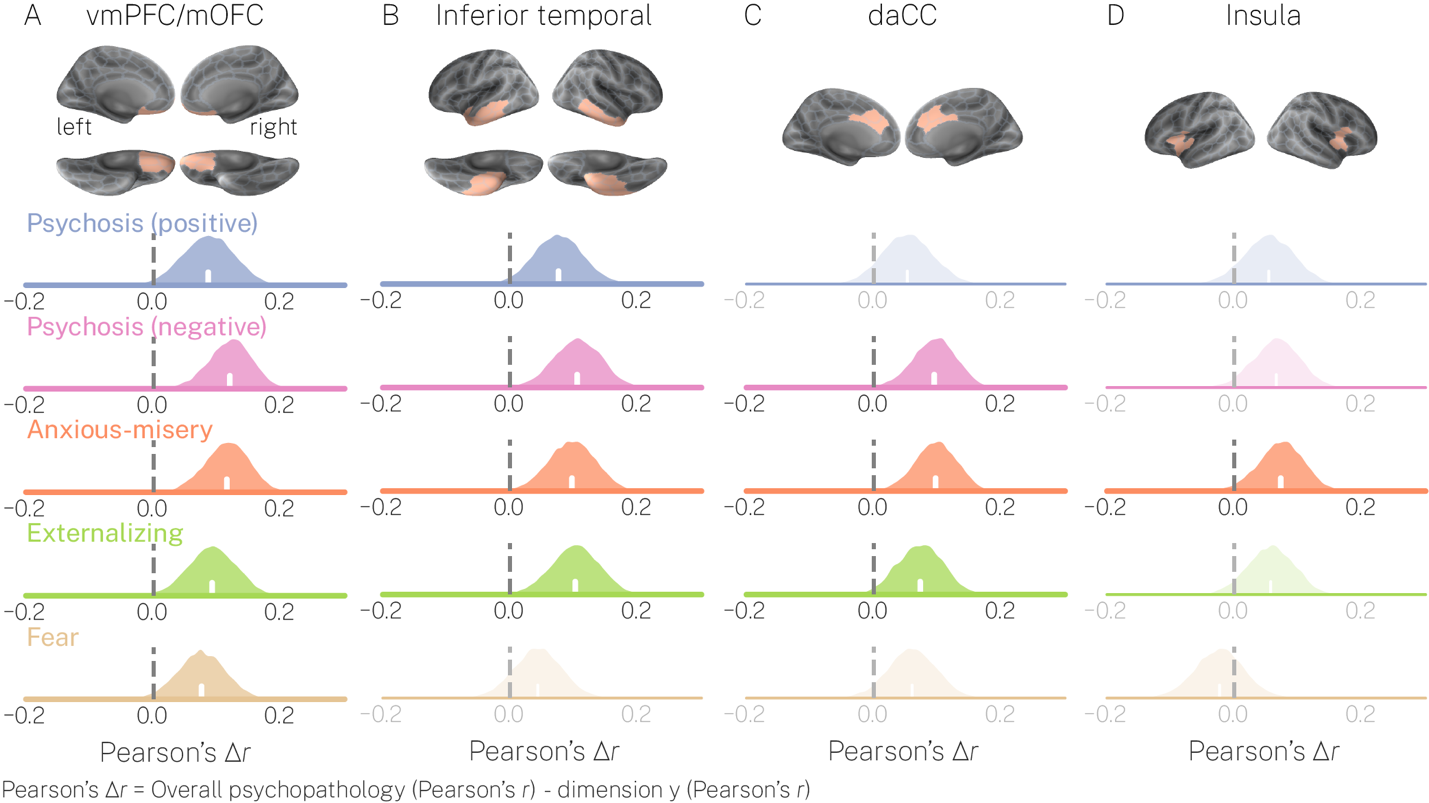
**

**Figure S16.** **Correlations between overall psychopathology and deviations from normative neurodevelopment are stronger than correlations observed for specific dimensions of psychopathology.** In each subplot, distributions of absolute Pearson’s correlations between each specific psychopathology dimension (rows) and regional deviations (columns) were subtracted from absolute correlations observed for overall psychopathology in the same region. Note, Pearson’s correlations were calculated after residualizing both psychopathology dimensions and deviations with respect to T1 QA and T1 SNR (see Supplementary Methods for details). Performing this subtraction 10,000 times across bootstrapped samples generated distributions of effect size differences, ∆*r*. Positive ∆*r* indicates that correlations for overall psychopathology were greater when compared to those observed for the specific dimensions. The ∆*r* distributions for which the lower bound of the *95*% confidence interval was greater than 0 are shown with heavier stroke and no transparency.

**REFERENCES:**

1 Satterthwaite TD *et al.* Neuroimaging of the Philadelphia Neurodevelopmental Cohort. *NeuroImage* 2014; **86**: 544–553.

2 Merikangas KR *et al.* Comorbidity of Physical and Mental Disorders in the Neurodevelopmental Genomics Cohort Study. 2015; **135**: 14.

3 Rosen AFG *et al.* Quantitative assessment of structural image quality. *NeuroImage* 2018; **169**: 407–418.

4 Schaefer A *et al.* Local-Global Parcellation of the Human Cerebral Cortex from Intrinsic Functional Connectivity MRI. *Cerebral Cortex* 2018; **28**: 3095–3114.

5 Daducci A *et al.* The Connectome Mapper: An Open-Source Processing Pipeline to Map Connectomes with MRI. *PLoS ONE* 2012; **7**: e48121.

6 Tustison NJ *et al.* Large-scale evaluation of ANTs and FreeSurfer cortical thickness measurements. *NeuroImage* 2014; **99**: 166–179.

7 Tustison NJ *et al.* N4ITK: Improved N3 Bias Correction. *IEEE Trans Med Imaging* 2010; **29**: 1310–1320.

8 Avants BB, Tustison NJ, Wu J, Cook PA, Gee JC. An Open Source Multivariate Framework for n-Tissue Segmentation with Evaluation on Public Data. *Neuroinform* 2011; **9**: 381–400.

9 Klein A *et al.* Evaluation of volume-based and surface-based brain image registration methods. *NeuroImage* 2010; **51**: 214–220.

10 Avants BB *et al*. A reproducible evaluation of ANTs similarity metric performance in brain image registration. *NeuroImage* 2011; **54**: 2033–2044.

11 Calkins ME *et al.* The Philadelphia Neurodevelopmental Cohort: constructing a deep phenotyping collaborative. *J Child Psychol Psychiatr* 2015; **56**: 1356–1369.
